# Discovery of a Novel Aminocyclopropenone Compound That Inhibits BRD4-Driven Nucleoporin NUP210 Expression and Attenuates Colorectal Cancer Growth

**DOI:** 10.3390/cells11030317

**Published:** 2022-01-18

**Authors:** Hiroya Kondo, Kenji Mishiro, Yuki Iwashima, Yujia Qiu, Akiko Kobayashi, Keesiang Lim, Takahiro Domoto, Toshinari Minamoto, Kazuma Ogawa, Munetaka Kunishima, Masaharu Hazawa, Richard W. Wong

**Affiliations:** 1 Division of Transdisciplinary Sciences, Graduate School of Frontier Science Initiative, Kanazawa University, Kakuma-machi, Kanazawa 920-1192, Japan; hiro4015@stu.kanazawa-u.ac.jp (H.K.); mishiro@p.kanazawa-u.ac.jp (K.M.); kogawa@p.kanazawa-u.ac.jp (K.O.); 2 Institute for Frontier Science Initiative, Kanazawa University, Kakuma-machi, Kanazawa 920-1192, Japan; akoba@staff.kanazawa-u.ac.jp; 3 Laboratory of Molecular Cell Biology, School of Natural System, Institute of Science and Engineering, Kanazawa University, Kakuma-machi, Kanazawa 920-1192, Japan; yuki090606@stu.kanazawa-u.ac.jp; 4 WPI-Nano Life Science Institute, Kanazawa University, Kakuma-machi, Kanazawa 920-1192, Japan; porthos0616@gmail.com (Y.Q.); limkeesiang@staff.kanazawa-u.ac.jp (K.L.); 5 Division of Translational and Clinical Oncology, Cancer Research Institute, Kanazawa University, Kakuma-machi, Kanazawa 920-0934, Japan; tdomoto@staff.kanazawa-u.ac.jp (T.D.); minamoto@staff.kanazawa-u.ac.jp (T.M.); 6 Faculty of Pharmaceutical Sciences, Institute of Medical, Pharmaceutical, and Health Sciences, Kanazawa University, Kakuma-machi, Kanazawa 920-1192, Japan; kunisima@p.kanazawa-u.ac.jp

**Keywords:** CRC, BRD4, NPC, NUP210, nuclear size, aminocyclopropenone, LLPS, IDR, MYC, cell growth

## Abstract

Epigenetic deregulation plays an essential role in colorectal cancer progression. Bromodomains are epigenetic “readers” of histone acetylation. Bromodomain-containing protein 4 (BRD4) plays a pivotal role in transcriptional regulation and is a feasible drug target in cancer cells. Disease-specific elevation of nucleoporin, a component of the nuclear pore complex (NPC), is a determinant of cancer malignancy, but BRD4-driven changes of NPC composition remain poorly understood. Here, we developed novel aminocyclopropenones and investigated their biological effects on cancer cell growth and BRD4 functions. Among 21 compounds developed here, we identified aminocyclopropenone 1n (ACP-1n) with the strongest inhibitory effects on the growth of the cancer cell line HCT116. ACP-1n blocked BRD4 functions by preventing its phase separation ability both in vitro and in vivo, attenuating the expression levels of BRD4-driven MYC. Notably, ACP-1n significantly reduced the nuclear size with concomitant suppression of the level of the NPC protein nucleoporin NUP210. Furthermore, NUP210 is in a BRD4-dependent manner and silencing of NUP210 was sufficient to decrease nucleus size and cellular growth. In conclusion, our findings highlighted an aminocyclopropenone compound as a novel therapeutic drug blocking BRD4 assembly, thereby preventing BRD4-driven oncogenic functions in cancer cells. This study facilitates the development of the next generation of effective and potent inhibitors of epigenetic bromodomains and extra-terminal (BET) protein family.

## 1. Introduction

Colorectal cancer (CRC) is the third leading cause of cancer-related mortality globally [1]. Unfortunately, after treatment, many CRC patients are susceptible to tumor recurrence and metastasis [2,3,4,5]. These clinical difficulties highlight the urgent need to develop effective treatment strategies as well as identify feasible therapeutic targets to improve patient mortality.

The nuclear pore complex (NPC), a cylindrical and symmetric microstructure composed of multiple copies of up to 30 different proteins termed nucleoporins (NUPs), is the sole gateway to the genome [6]. Using high-speed atomic force microscopy (HS-AFM), we recently revealed that the native nuclear pore inner channel resembles hydrogel cobwebs and achieves liquid–liquid phase separation (LLPS) in HCT116 colon cancer cells and organoids [7,8]. Clinically, we also showed that nucleoporin TPR expression was increased in CRC databases and primary tumors of CRC patients [9,10,11,12]. We and others also demonstrated that the expression of nuclear pore proteins is vital for maintaining genomic integrity, and suggested that alterations in their roles could contribute to changes in nucleus size and cancer development [13]. Indeed, several transcription enhancers tether transcriptionally active loci to the NPC, and also promote large-scale gene–NPC interactions in cancer cells [14], highlighting the direct correlation among enhancers–chromatin–NPC [15], which also provides potential therapeutic targets for CRC.

Super-enhancers (SEs) are clusters of transcription enhancers that drive gene expression. They are normally characterized by high levels of acetylation of histone H3 lysine 27 (H3K27ac), which is catalyzed by the histone lysine acetyltransferase CREB binding protein (CBP). Cancer cells often acquire tumor-specific SEs at vital oncogenes, such as MYC, which induce several hallmarks of cancer. Bromodomain-containing protein 4 (BRD4), a member of the bromodomains and extra-terminal (BET) protein family [16,17,18,19], binds to acetylated H3 lysine 27 (H3K27ac) on chromatin, where transcription regulators are recruited to form multi-molecular assemblies [20,21]. BRD4 is recruited to SEs and consequently functions as an epigenetic reader to promote the transcription of SE-marked genes in cancer cells. Thus, the role of BRD4 in cancers is recognized to involve the regulation of SE organization and oncogene expression [19]. In this regard, a number of epigenetic agents have been developed, including inhibitors of BET family proteins (JQ1, ARV-771) [22,23]. Many findings have suggested that BET inhibitors display promising anti-cancer effects through perturbing transcriptional regulation, most notably SEs [24]. Recent findings have suggested that phase-separated multi-molecular condensates occur during SE establishment, in which interactions mediated via intrinsically disordered regions (IDRs) of coactivators, such as BRD4 and transcription factors (TFs) play critical roles [25]. The multivalent interactions among IDRs promote LLPS, where the highly concentrated transcriptional mechanism ensures the robust expression of oncogenes [26]. In this regard, targeting the phase separation ability of BRD4 may be a novel strategy for treating cancer. Furthermore, different small-molecule inhibitors of BRD4 have recently been developed, such as JQ1, because transcription of the MYC oncogene is dependent on BRD4. JQ1 occupies the bromodomain pockets of BRD4, preventing its binding to acetylated histones and selectively repressing transcription of the MYC oncogene and MYC-dependent genes.

Moreover, the progression of CRC development is also dependent on aberrant MYC expression [27]. CRCs are also known to be the carcinomas most characterized by histone acetylation [28]. Thus, we focused on BRD4 as BRD4, which is involved in MYC expression and histone acetylation, which can be proposed as a potential therapeutic target for CRC [29,30]. Targeting BRD4 thus represents a promising strategy for treating CRC.

Here, we developed a series of aminocyclopropenone compounds and explored their biological effects on IDR-related functions of BRD4 [31,32]. The present study demonstrated that a novel aminocyclopropenone compound inhibits the assembly of BRD4, thus preventing BRD4-driven oncogenic transcription. Moreover, we identified nucleoporinNUP210 as an NUP upregulated in a BRD4-dependent manner, which contributes to cancer cell growth and maintenance of the nuclear architecture in CRC.

## 2. Materials and Methods

### 2.1. Cell Culture

HEK293T, SAS, SW480, SW620, and HCT116 cells were maintained in Dulbecco’s Modified Eagle’s Medium (DMEM) supplemented with 10% (vol/vol) fetal bovine serum (FBS) and 1% (vol/vol) penicillin/streptomycin (P/S). ccd18co cells were maintained in Modified Eagle’s Medium (MEM) with 10% (vol/vol) fetal bovine serum (FBS) and 1% (vol/vol) penicillin/streptomycin (P/S). These cells were incubated at 37 °C and 5% CO_2_ in a humidified atmosphere.

### 2.2. Western Blotting

Cells were lysed with lysis buffer [52.5 mM Tris-HCl (pH6.8), 2.15% sodium dodecyl sulfate, 5% β-mercaptoethanol, 7% glycerol, and 0.0025% bromophenol blue]. Cell lysates were subjected to SDS-PAGE followed by conventional wet transfer. Membranes were incubated with antibodies and exposed to secondary horseradish peroxidase-conjugated antibodies (Cell Signaling Technology, Danvers, MA, USA, and Invitrogen, Waltham, MA, USA). Images were detected using an LAS-4000 image analyzer (Fujifilm, Tokyo, Japan). Antibodies used in this study are listed in Supplementary Table S1.

### 2.3. Cell Proliferation Assay

Cells were seeded into 96-well plates at a density of 3000 cells per well and cultured for the indicated times. Cell viability was assessed using the 3-(4,5-dimethylthiazol-2-yl)-2,5-diphenyltetrazolium bromide (MTT; Tokyo Chemical Industry, Tokyo, Japan; D0801) method. Briefly, 10 μL of 12 mM MTT solution was added to each well, incubated for 3 h, and the reaction was stopped by adding 100 μL of STOP solution [2% acetic acid, 16% SDS (Wako, Osaka, Japan; 194-13985), and 42% *N*, *N*-dimethyl formamide (Nakalai Tesque, Kyoto, Japan; 13016-65)], as previously described [13]. Samples were mixed thoroughly and measured at 570 nm for absorbance.

### 2.4. Colony Formation Assay

Cells were seeded into 3 cm dishes at 300 cells/dish and cultured for 10 days, followed by staining with crystal violet.

### 2.5. cDNA Preparation and Quantitative Real-Time RT-PCR Assay

We used 500 ng of RNA for cDNA preparation using ReverTra Ace^®^ qPCR RT Master Mix (TOYOBO, Osaka, Japan). Quantitative real-time RT-PCR was performed using SYBR^®^ Premix Ex Taq™ II (Takara, Shiga, Japan) in a Thermal Cycler Dice^®^ Real Time System (Takara, Shiga, Japan), in accordance with the manufacturer’s instructions. The relative mRNA expression levels of target genes were calculated using GAPDH as a loading control. Primer sequences are listed in Supplementary Table S2.

### 2.6. Immunofluorescence Analysis

Cells were cultured on glass coverslips in 12-well plates. Cells on coverslips were washed in phosphate-buffered saline [PBS; 137 mM NaCl (Nacalai Tesque, Kyoto, Japan; 31320-34), 2.7 mM KCl (Sigma-Aldrich, St. Louis, USA; P9541), 10 mM Na2HPO4 (Wako, Osaka, Japan; 198-05955F), and 1.8 mM KH2PO4 (Nacalai Tesque, Kyoto, Japan; 28736-75)], fixed for 20 min with 4% paraformaldehyde (Nacalai Tesque, Kyoto, Japan; 26123-55) in PBS, washed again with PBS, and permeabilized with 0.3% Triton X-100 (Nacalai Tesque, Kyoto, Japan; 35501-15) in PBS for 3 min. Cells were then washed and blocked in PBS containing 4% bovine serum albumin (BSA; Wako, Osaka, Japan; 015-23295) for 30 min at room temperature. Coverslips were then incubated overnight in 4% BSA/PBS containing primary antibodies (1:200 dilution). Subsequently, cells were rinsed and incubated with secondary antibodies in 4% BSA/PBS (1:200 dilution) for 1 h at room temperature. After washing with PBS, coverslips were mounted on slides using ProLong Diamond Antifade reagent with DAPI (Invitrogen, Waltham, MA, USA; P36966), and observed on a confocal laser-scanning microscope with a 60× PlanApo/1.45NA DIC objective (Olympus, Tokyo, Japan; FV10i-LIV), as previously described [33].

### 2.7. Transfections, Viral Particle Production, and Infection

DNA transfections were performed using Lipofectamine 2000. Lentiviral particles were produced with the MISSION Lentiviral Packaging System (Sigma-Aldrich, St. Louis, MO, USA). HCT116 cells were transduced with the lentiviral particles in the presence of 8 μg/mL polybrene (Sigma-Aldrich, St. Louis, MO, USA) for 48 h.

### 2.8. Data Analysis for ChIP-Seq

The ChIP-seq data for input, H3K27ac, and BRD4 in the HCT116 cell line were downloaded from GSE73319. ChIP-seq reads were aligned to the hg19 genome assembly using Bowtie2 with the default parameters. Only tags that uniquely mapped to the genome were used for further analysis. PCR duplicates were removed using Picard tools (http://broadinstitute.github.io/picard, accessed on 12 January 2022). Peaks were identified with the homer findPeaks.pl script. Peak annotation was processed with the homer annotatePeaks.pl script. Heatmaps were plotted using deeptools. Bigwig files were generated using the deepTools bamCoverage function with—normalizeUsing RPGC—effectiveGenomeSize 2864785220—binSize 1. Peak annotation and bed files were obtained using HOMER (http://homer.ucsd.edu/homer/ngs/, accessed on 12 January 2022). Bed files of SE in HCT116 were obtained from SEdb (http://www.licpathway.net/sedb/index.php, accessed on 12 January 2022). Heatmap analysis was performed using ngs.plot [34].

### 2.9. Nuclear Size Measurement

Nuclear size measurements were normalized to controls. Statistical analysis was performed by summing up all independently repeated experiments. The nuclear size was calculated by multiplying the major axis and the minor axis of the nucleus. Two-tailed Student’s *t*-test assuming equal variances was performed using GraphPad Prism 7 software to evaluate statistical significance. *p*-values, number of nuclei quantified, and error bars are denoted in the figure legends.

### 2.10. In Vitro Droplet Assay

Recombinant mEGFP protein was obtained from ORIGENE (Sku#TP790050). Recombinant mEGFP fusion protein (BRD4aa674–1351; BRD4-IDR–mEGFP) was generated as described previously [35] and concentrated to an appropriate level using Amicon Ultra centrifugal filters (50K MWCO, Millipore, Burlington, MA, USA). Recombinant protein was diluted to a final concentration of 5 μM in droplet buffer (50 mM Tris–HCl pH 7.5, 10% glycerol, 1 mM DTT, and 10% PEG) containing 10 µM of each aminocyclopropenone, and then immediately loaded on a glass-bottomed dish (MATSUNAMI) and covered with a coverslip. Samples were imaged using a Zeiss LSM5 EXCITER microscope with a mercury lamp and a Plan-Apochromat 100×/1.4 oil objective, as previously described [35]. Axio Vision software (version 4.8) was used for image acquisition.

### 2.11. Statistical Analysis

Cell proliferation assay, colony formation assay, western blotting, nuclear size measurement, in vitro droplet assay, and real-time RT-PCR analysis were all performed in triplicate (*n* = 3) and independently replicated three times. Data are the mean of independent experiments (*n* = 3). Statistical analyses were performed using GraphPad Prism software. Nuclear size measurement is presented as mean ± standard deviation, while all other data are presented as mean ± standard error. Statistically significant differences in mean or median values between respective groups were tested by Student’s *t*-test or Mann–Whitney test. *p* values < 0.05 were considered to indicate a statistically significant difference. When the control group is considered as 100%, one-sample *t*-test was performed using GraphPad QuickCalcs (http://graphpad.com/quickcalcs/OneSampleT1.cfm, accessed on 12 January 2022).

## 3. Results

### 3.1. The Effects of Aminocyclopropenone Compounds on Colorectal Cancer Cell Growth and in Vitro Droplets of BRD4

Cyclopropenones are cyclic enones that possess a strained three-membered ring. Because of their unique structure, they could be promising building blocks for drug discovery. However, the bioactivity of cyclopropenone derivatives has rarely been explored, and only a few reports investigate bioactive cyclopropenones [36,37,38]. Additionally, cyclopropenones have a unique photochemical property. Photoexcitation releases the ring strain of cyclopropenones to produce an alkyne and carbon monoxide. This photochemical alkyne generation is useful for generating highly reactive alkynes, such as cyclooctyne and ynamine, and conducting chemical reactions using these alkynes in controlled space and time. Recently, we reported photochemical reactions using aminocylopropenones [31,32]. 

**Figure 1:**
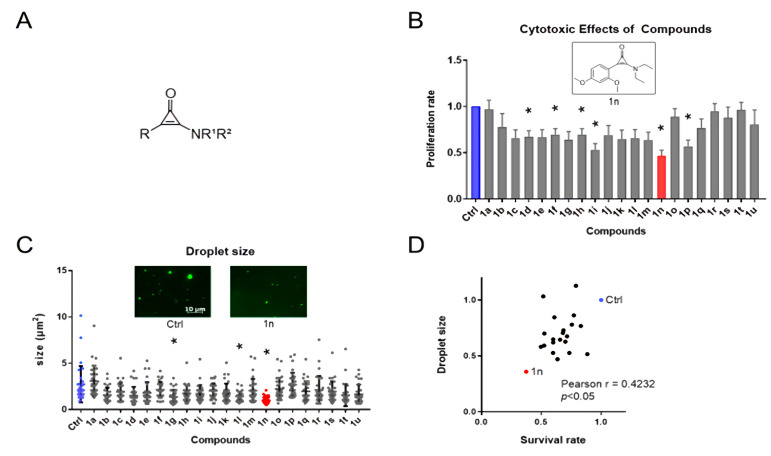
Aminocyclopropenone 1n inhibits colorectal cancer cell growth and LLPS formation. (**A**) Basic skeletal structure of aminocyclopropenone compounds. (**B**) Relative viable number of HCT116 cells after treatment with a series of aminocyclopropenones (72 h, 10 µM); structure of the 1n compound is also presented. The proliferation rate of control HCT116 cells is set as 1.0. Data show mean ± SE from three independent experiments (*n* = 3) and * indicates *p* < 0.05. (**C**) Relative size of BRD4 droplets after treatment with a series of aminocyclopropenones (10 µM) and images of representative BRD4 droplets after 1n treatment are presented. * indicates *p* < 0.0001. (**D**) Plot showing the correlation between cell growth inhibitory effects on HCT116 colorectal cancer cells (72 h, 10 µM) and in vitro BRD4 droplet formation under a series of aminocyclopropenone treatments.

In these reactions, an aminocyclopropenone is photolyzed to produce an ynamine, which acts as a potent dehydration condensation agent. This reaction may be applicable for determining the drug targets of bioactive compounds containing an aminocyclopropenone. In contrast, to the best of our knowledge, the bioactivity of amino-substituted cyclopropenones has never been explored, and the biological properties of aminocyclopropenones remain completely unknown. Because an aminocyclopropenone is a kind of vinylogue of an amide, a carbonyl oxygen of an aminocyclopropenone could work as a hydrogen bond acceptor. Additionally, a carbon atom of a carbonyl group and two carbon atoms of a double bond conjugated with the carbonyl group have electrophilic nature and might interact with a nucleophile. Due to these characters, an aminocyclopropenone is expected to interact with various amino acid residues in a protein by hydrogen bonds and electrostatic interactions. In this study, to extend the versatility of aminocyclopropenones, we explored the biological effects of aminocyclopropenones on IDR and/or LLPS. We synthesized a series of aminocyclopropenone compounds (Figure 1A and Figure S1) and initially investigated their growth inhibitory effects against the colorectal cancer cell line HCT116.

Among 21 compounds, aminocyclopropenone 1n (ACP-1n) (~90% purity) showed the strongest anti-proliferative effect (Figure 1B). Next, we investigated whether aminocyclopropenone compounds attenuate the phase separation ability of BRD4, which is a determinant of the aberrant transcriptional activity underlying cancer cell growth. We generated IDRs of BRD4 fused with mEGFP and evaluated the phase separation ability for each aminocyclopropenone compound. In vitro droplet formation assay demonstrated that ACP-1n is the most potent inhibitor of BRD4-IDR assembly (Figure 1C and Figure S2). Notably, the effects of inhibiting phase separation were positively correlated with the anti-growth activities (Figure 1D). These results indicate that ACP-1n can suppress cancer cell growth by preventing BRD4 assembly.

### 3.2. Aminocyclopropenone Compound ACP-1n Prevented Super-Enhancer-Driven MYC Expression by Blocking BRD4 Assembly in the Nucleus

**Figure 2:**
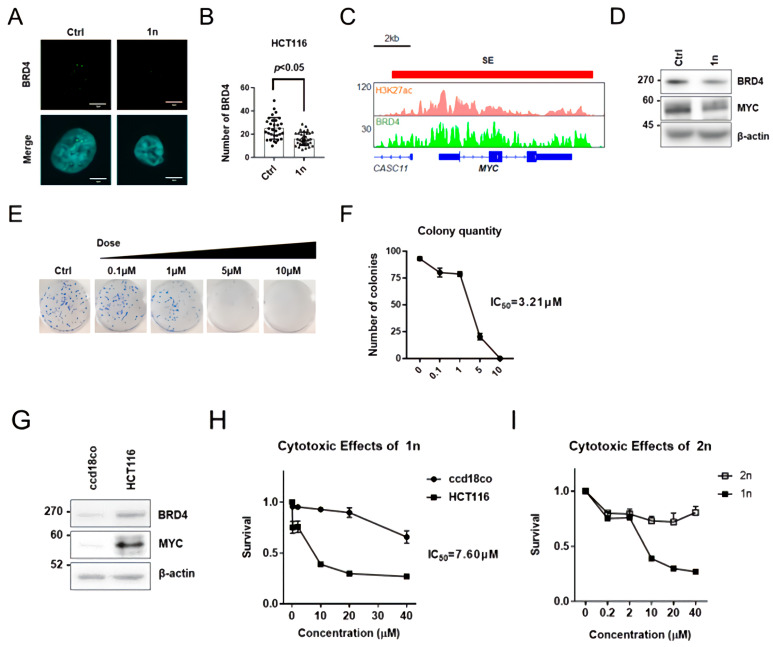
Aminocyclopropenone 1n reduces the expression level of the BRD4-driven oncogene MYC. (**A**) Immunofluorescence confocal microscopic analysis of BRD4 in HCT116 cells after aminocyclopropenone 1n treatment (12 h, 10 μM). (**B**) Number of BRD4 aggregates in the nucleus upon aminocyclopropenone 1n administration (12 h, 10 μM). (**C**) Gene tracks of H3K27ac and BRD4 ChIP-seq occupancy at representative super-enhancer-associated genes in colorectal cell. The x-axis shows genomic position and the y-axis shows signal of ChIP-seq occupancy in units of reads per million mapped reads per base pair (rpm/bp). (**D**) Western blotting analysis of BRD4 and MYC levels upon aminocyclopropenone 1n administration (24 h, 10 µM) in HCT116 cells. (**E**) Long-term proliferation assay of HCT116 cells under different aminocyclopropenone 1n concentrations. (**F**) Quantification of HCT116 colonies under different aminocyclopropenone 1n concentrations. Data show mean ± SE from three independent experiments (*n* = 3). (**G**) Western blotting analysis of BRD4 and MYC levels in HCT116 and ccd18co cells. (**H**) Effect of aminocyclopropenone 1n on viability of HCT116 and ccd18co cells. Data show mean ± SE from three independent experiments (*n* = 3). (**I**) Proliferation of HCT116 cells under different concentrations of 1n and 2n examined by MTT assay. Data show mean ± SE from three independent experiments (*n* = 3).

We next elucidated the functions of ACP-1n against cancer growth [13,35,39,40] and its functional significance in the maintenance of CRC in vitro by targeting BRD4 and BRD4-driven gene expression in colorectal cancer HCT116 cells. Consistent with the previous results from an in vitro droplet formation assay (Figure 1C), immunofluorescent confocal imaging of BRD4 proteins demonstrated that the numbers of BRD4 puncta (representing BRD4 assembly) significantly decreased following ACP-1n treatment (Figure 2A,B). To further explore the potential inhibitory function of BRD4 occupancy, we first analyzed the genome-wide interaction and gene tracks of H3K27ac and BRD4 ChIP-seq occupancy at super-enhancer-associated genes in HCT116 cells. In agreement with recent reports, MYC, known as a BRD4-driven oncogene, was found (Figure 2C). 

As expected, ACP-1n-treated cells showed lower levels of MYC, one of the SE-driven oncogenes, than controls (Figure 2D). We next examined whether ACP-1n is functionally involved in the proliferation as well as the viability of HCT116 cells using various concentrations of ACP-1n (Figure 2E,F). In HCT116 cells, increasing the concentration of ACP-1n decreased the clonogenic potential to 50% at 3.21 µM. Hence, the anti-growth ability of ACP-1n was specific to HCT116 cells. Notably, the expression levels of both BRD4 and MYC were elevated in HCT116 cancer cells compared with those in the normal colorectal cell line ccd18co (Figure 2G). Because ACP-1n inhibited the growth of HCT116 cells and downregulated BRD4 and MYC expression (Figure 2D), we next examined any effects of ACP-1n on the growth of normal colon cells. Remarkably, we found that ACP-1n had a negligible inhibitory effect on the proliferation of normal colon cells. Hence, the anti-proliferative effect of ACP-1n was specific to colorectal HCT116 cancer cells (Figure 2H). We also prepared an ACP-1n analog without the cyclopropenone structure (2n) and evaluated its effects on cell growth and proliferation. We found that 2n lacked anti-proliferative effects (Figure 2I), indicating that the cyclopropenone structure plays an essential role in inhibiting BRD4 functions through perturbing phase separation, thereby suppressing cancer cell growth. Collectively, these findings indicate that ACP-1n potentially suppressed super-enhancer-driven MYC expression by blocking BRD4 assembly in the nucleus of HCT116 cells in vitro.

### 3.3. Identification of NUP210 as a BRD4-Driven Nuclear Complex Component

**Figure 3:**
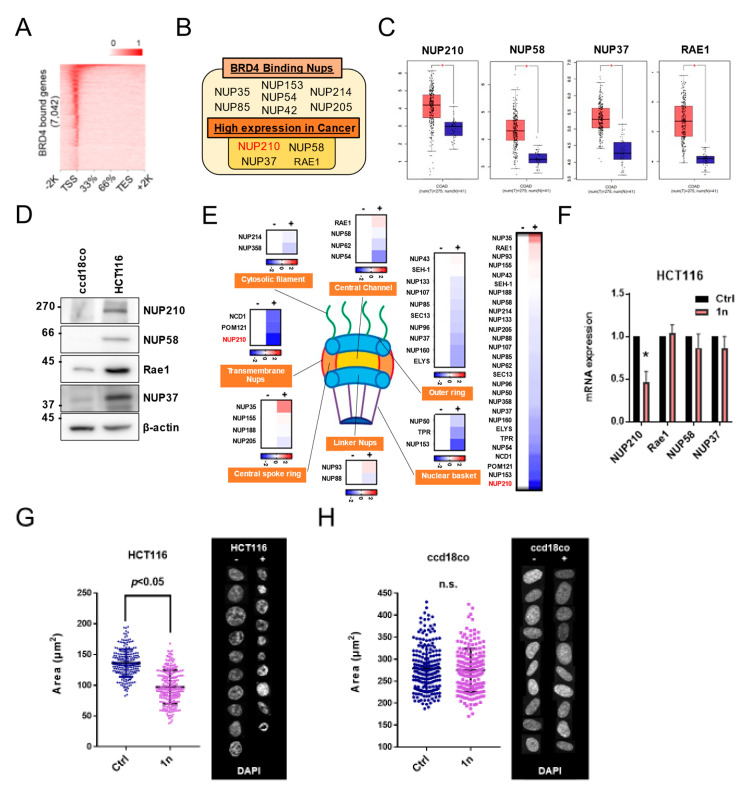
Aminocyclopropenone 1n reduces the nucleus size of colorectal cancer cells. (**A**) Heatmap of BRD4 ChIP-seq peak enrichment over gene bodies in HCT116. (**B**) Venn diagram illustrating the overlap between BRD4-binding NUPs and those with high expression in colorectal cancer. (**C**) The expression of NUP210, NUP58, NUP37, and RAE1 in colorectal cancer (GEPIA). (**D**) Western blotting analysis of NUP210, NUP58, NUP37, and Rae1 levels in HCT116 and ccd18co cells. (**E**) Heatmap representing NUP transcript levels in HCT116 cells after aminocyclopropenone 1n treatment (24 h, 10 µM). (**F**) qRT-PCR analysis of candidate nucleoporin gene mRNA in HCT116 cells after aminocyclopropenone 1n treatment (24 h, 10 μM). Expression level of mRNA from control HCT116 cells is set as 1.0. Data show mean ± SE from three independent experiments (*n* = 3) and * indicates *p* < 0.05. (**G**) Montages of representative DAPI-stained nuclei and measurement of the nucleus size after treatment with 1n (24 h, 10 µM) in HCT116 cells. Statistical analysis was performed by summing up all independently repeated experiments (*n* = 3). (**H**) Montages of representative DAPI-stained nuclei and measurement of the nucleus size after treatment with aminocyclopropenone 1n (24 h, 10 µM) in ccd18co cells. Statistical analysis was performed by summing up all independently repeated experiments (*n* = 3).

Quantitative and qualitative changes of nuclear transport proteins and nucleoporins are determinants of cancer cell growth and malignant phenotypes [41]. Hence, we next characterized disease-specifically overexpressed nucleoporins/NUPs that are potently induced by BRD4. We performed chromatin immunoprecipitation sequencing (ChIP-seq) with an antibody against BRD4 (Figure 3A). The majority of BRD4 peaks were in intergenic regions, while the rest were found predominantly within gene bodies, including exons and introns. Furthermore, BRD4 tended to accumulate on the transcription start site (TSS) of genes. Interestingly, out of 7042 genes screened, we found that one-third of NUPs are potent targets of BRD4 (Figure 3B). We further used the online database Gene Expression Profiling Interactive Analysis (GEPIA) to determine NUPs aberrantly expressed in colorectal cancer tissue. The transcript levels of four NUPs (NUP210, NUP58, NUP37, and Rae1) were higher in colon cancer tissue among BRD4-bound NUPs (Figure 3C). Consistent with these publicly available data, the protein level of each NUP was much higher in HCT116 cells than in ccd18co cells (Figure 3D). Moreover, to investigate whether these changes in NUPs were involved in ACP-1n-treated cells, we performed qRT-PCR to evaluate the mRNA levels following ACP-1n treatment (Figure 3E). We found that NUP210 was most strongly inhibited by ACP-1n (Figure 3F). Because NUPs are the key gatekeepers and determinants of nuclear architecture, we further evaluated the morphological changes. We were prompted to further explore the effect of ACP-1n on the control of nucleus size. Notably, ACP-1n significantly reduced the nuclear size of the colorectal cancer cells HCT116 (Figure 3G), but not normal colorectal cells ccd18co (Figure 3H). These findings also indicated that ACP-1n alters the epigenomic features of BRD4-driven NUP210, so regulating nucleus size could be involved in cancer cell growth.

### 3.4. The Expression Levels of NUP210 Determine Cancer Cell Growth and Nucleus Size

**Figure 4:**
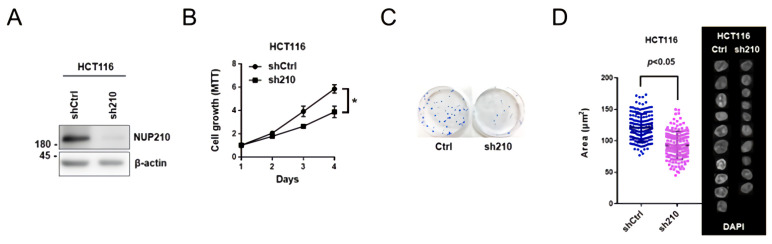
Expression level of NUP210 adjusts the proliferative capacity and nucleus size of colorectal cancer cells. (**A**) Western blotting analysis of NUP210 levels in HCT116 cells expressing shRNA NUP210. (**B**) Proliferation of NUP210-silenced HCT116 cells examined by MTT assay. Data show mean ± SE from three independent experiments (*n* = 3) and * indicates *p* < 0.05. (**C**) Long-term proliferation assay of HCT116 cells expressing shRNA NUP210. (**D**) Montages of representative DAPI-stained nuclei and measurement of the nucleus size in HCT116 cells expressing shRNA NUP210. Statistical analysis was performed by summing up all independently repeated experiments (*n* = 3).

To evaluate whether NUP210 is functionally involved in colon cancer cells, we investigated the growth ability as well as nuclear morphology of HCT116 cells stably expressing shRNA targeting NUP210 (Figure 4A). Both short-term and long-term proliferation assays demonstrated that in NUP210-depleted HCT116 cells, cell growth ability was impaired (Figure 4B,C). Additionally, NUP210 silencing resulted in a decrease of nucleus size (Figure 4D), as observed in ACP-1n-treated cells. Taken together, these findings show that modulating NUP210 levels affects nucleus size and cell growth, implying associated changes in nuclear import capacity, NPC numbers, and the localization of nuclear transport factors and lamins.

### 3.5. ARV-771, IPZ, U0126, and LY294002 Are Unable to Inhibit In Vitro BRD4 Assembly

**Figure 5:**
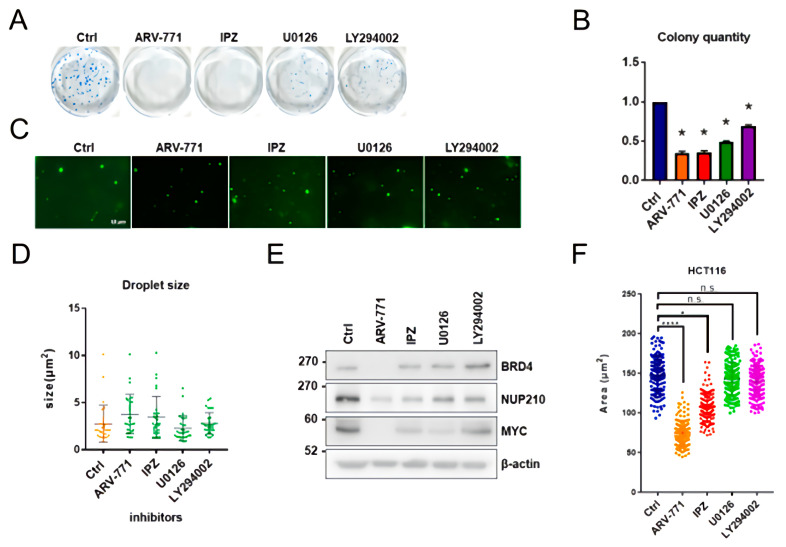
ARV-771, IPZ, U0126, and LY294002 cannot inhibit in vitro BRD4 assembly. (**A**) Long-term proliferation assay of HCT116 cells with ARV-771, IPZ, U0126, and LY294002 inhibitors (10 µM). (**B**) The quantification of HCT116 colonies with ARV-771, IPZ, U0126, and LY294002 inhibitors. The number of colonies from control HCT116 cells is set as 1.0 and * indicates *p* < 0.05. (**C**) Representative images of BRD4 droplets after treatment with ARV-771, IPZ, U0126, and LY294002 inhibitors. (**D**) Relative size of BRD4 droplets after treatment with ARV-771, IPZ, U0126, and LY294002 inhibitors (10 µM). (**E**) Western blotting analysis of BRD4, NUP210, and MYC levels in HCT116 cells after treatment with ARV-771 (24 h, 10 µM), IPZ (24 h, 20 µM), U0126 (24 h, 10 µM), and LY294002 (24 h, 10 µM). (**F**) Measurement of the nucleus size after treatment with ARV-771 (24 h, 10 µM), IPZ (24 h, 20 µM), U0126 (24 h, 10 µM), and LY294002 (24 h, 10 µM) in HCT116 cells. Statistical analysis was performed by summing up all independently repeated experiments (*n* = 3) and * indicates *p* < 0.05, while **** indicates *p* < 0.0001.

Anti-cancer drugs, including ARV-771 [pan-(bromodomain and extra-terminal) BET degrader], IPZ (importazole, importin-β inhibitor), U0126 (selective inhibitor of the MAP kinase kinases MEK1 and MEK2), and LY294002 (PI3-kinase inhibitor) reduced HCT116 cell growth (Figure 5A,B). Consistent with previous reports, treatment with a BET inhibitor (ARV-771) also inhibited MYC expression levels by degrading BRD4 [42]; however, ARV-771 was unable to inhibit in vitro BRD4-IDR condensates (Figure 5C). Notably, IPZ, U0126, and LY294002 were also unable to inhibit in vitro BRD4 assembly (Figure 5C,D), while ARV-771 strongly inhibited both NUP210 expression and nuclear size (Figure 5E,F). This also suggests that the decrease in NUP210 expression results in a reduction in nucleus size.

**Figure 6:**
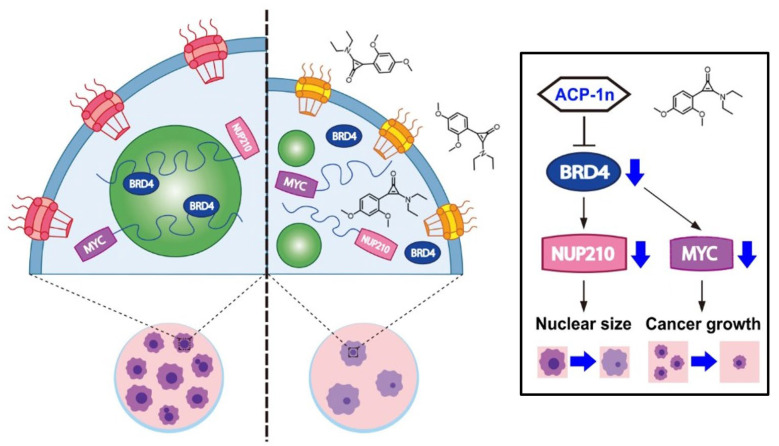
Schematic working model. ACP-1n inhibits BRD4 assembly and blocks the expression of oncogene MYC and nuclear size regulator NUP210. As a result, it reduces the proliferative capacity and nuclear size of colorectal cancer.

Furthermore, we also confirmed two colorectal cancer cell lines (SW480 and SW620) and one non-colorectal cancer cell line, SAS (from human head and neck squamous cell carcinoma (HNSCC)) to ensure the major observations (growth inhibitory testing, BRD4 LLPS formation, proliferation assays) by ACP-1n were not just specific to HCT116 cells (Figures S3 and S4). Meanwhile, we also investigated whether downregulation of BRD4 or decreased LLPS of BRD4 was the cause of BRD4 downstream gene changes. As shown in the Figure S5, the protein expression of BRD4 was not significantly changed after ACP-1n treatment (12 h, 10 μM) in several CRC cell lines, but there was significant reduction in the numbers of BRD4 puncta (representing BRD4 assembly) following ACP-1n treatment (12 h, 10 μM) (Figure 2A); these data suggest that ACP-1n specifically targets BRD4 phase separation in vitro. We also found that ACP-1n treatment did not affect the transcription of BRD4 (Figure S6). Finally, we also compared ACP-1n to a histone deacetylase inhibitor (Trichostatin A [TSA]) (Figure S7). Although TSA was more effective in reducing the proliferative capacity of cancer cells than ACP-1n, the vending point of our novel compound (ACP-1n) which is specific targeting in phase separation aggregates and is also capable of reducing proliferative capacity. Together, our results suggest that BRD4-driven NUP210 regulates cancer cell growth and nuclear architecture. Unlike other existing inhibitors (ARV-771, IPZ, U0126, and LY294002), the aminocyclopropenone ACP-1n, as a novel inhibitor of LLPS formation, induced decreases in nucleus size and CRC cell proliferation via epigenetic pathways associated with BRD4 occupancy (Figure 6).

## 4. Discussion

Abnormalities in genetic and epigenetic modifications can induce drastic changes in gene expression profiles that are linked to various cancer types. The NPC and its 30 different NUPs have also been viewed as a nuclear architectural platform that influences genome function and gene expression by mediating spatial and temporal coordination among transcription factors, chromatin regulatory proteins, and transcriptional machinery [43]. These transcription factors create and engage super-enhancers (SEs) by recruiting acetylation writers depositing permissive H3K27ac chromatin marks. These SEs are strongly associated with BET proteins, including BRD4, that influence higher-order chromatin structure. The orchestration of these events triggers accessibility of RNA polymerase machinery and the imposition of lineage-specific gene expression [44]. Therefore, if we can directly manipulate the IDR SEs–chromatin–NUPs interactions, this could provide a novel therapeutic target in cancer.

Here, we developed a novel bioactive aminocyclopropenone compound and evaluated its biological properties against colorectal cancer cells. Notably, ACP-1n showed the ability to suppress BRD4 assembly, resulting in the prevention of cancer cell growth with simultaneous reduction in nucleus size. We also demonstrated that the nucleoporin NUP210 was overexpressed and its expression was driven by BRD4, which regulated the proliferative ability and nuclear architecture of colorectal cancer cells. Thus, we identified novel biological activity of the aminocyclopropenone in preventing IDR BRD4 function, thereby contributing to the suppression of BRD4-driven oncogenic signaling.

Various lines of evidence have suggested that BRD4 facilitates malignant phenotypes in diverse cancer contexts. In particular, as an epigenetic reader of the histone code, BRD4 recognizes H3K27ac through the BET domain and recruits transcriptional regulators to establish SEs [45]. To date, many small-molecule BRD4 inhibitors have been discovered, some of which are in clinical trials for the treatment of different diseases. For example, inhibitors of BET family proteins (JQ1, ARV-771) are promising anticancer agents that act through perturbing multiple components involved in transcriptional regulation. However, results have revealed that most of them have certain toxic side effects [46]. Thus, there is an urgent need for novel strategies targeting BRD4 to enhance the biological activity of these inhibitors and reduce toxic side effects. In this report, we have shown that ACP-1n inhibited BRD4 functions by targeting its phase separation ability. Recent research has demonstrated that the phase-separating properties of IDRs in TFs and BRD4 underlie the compartmentalization and concentration of transcriptional components, including SEs. Therefore, targeting networks of weak protein–protein interaction involved in phase-separated molecular condensates could provide novel therapeutic strategies.

Here, we designed and synthesized a cyclopropenone compound, ACP-1n. The use of photo-ACP-1n to study epigenetic activation is a focus of current studies in our lab.

Alteration of nuclear architecture is frequently observed in cancer cells. In this study, we also demonstrated that preventing BRD4 functions reduced not only the excessive growth capacity of cancer cells but also the size of their nucleus. Regarding the mechanism involved in this, we also identified NUP210 as a BRD4-driven NUP and determinant of the nuclear architecture in colorectal cancer cells. Interestingly, Jevtić et al. also reported that the expression level of another nucleoporin, ELYS, controls nucleus size in mammalian MCF10A cells [47]. Notably, ACP-1n did not affect the expression of other NUPs in our preliminary screen, and future work will focus on how ACP-1n changes transcriptionally and whether chromatin structure affects nucleus size in rodents.

In summary, our results demonstrated that the aminocyclopropenone ACP-1n inhibits LLPS formation in colorectal cancer cells, causing dramatic reductions in cell growth and nucleus size. We also identified the MYC oncogene and NUP210, an NPC component, as factors associated with these effects, opening a new avenue for generating epigenetic IDR inhibitors. In this context, it is anticipated that targeting non-structure-dependent phase separation formation will become a promising therapeutic approach for cancer therapy.

## Data Availability

Data is contained within the article or Supplementary Materials.

## Supplementary Materials

The following supporting information can be downloaded at: https://www.mdpi.com/article/10.3390/cells11030317/s1. Figure S1: Synthetic procedure and NMR spectra of aminocyclopropenone compounds. Figure S2: Representative BRD4 droplets pictures after each aminocyclopropenone treatment. Figure S3: Aminocyclopropenone 1n inhibits SW480 and SW620 colorectal cancer cells growth and in vivo BRD4 assembly respectively. Figure S4: Aminocyclopropenone 1n also inhibits human head and neck squamous cell carcinoma (HNSCC) SAS cells growth and in vivo BRD4 assembly. Figure S5: No visible changes of BRD4 protein expression after ACP-1n treatment (12 h, 10 μM) in several CRC cell lines. Figure S6: qRT-PCR analysis of BRD4 mRNA in HCT116 cells after aminocyclopropenone 1n treatment (24 h, 10 μM). Figure S7: Effect of trichostatin A on viability of HCT116 cells. Table S1: Antibodies used in this study. Table S2: Primers sequences for qRT-PCR.
