# Supplementary material for: Discovery of a Novel Aminocyclopropenone Compound That Inhibits BRD4-Driven Nucleoporin NUP210 Expression and Attenuates Colorectal Cancer Growth
Source: Cells. 2022 Jan 18;11(3):317. doi: 10.3390/cells11030317 (PMC8833887; doi:10.3390/cells11030317)
Supplement: Supplementary file 1 [file cells-11-00317-s001.zip › Supplementary Figure S1.pdf]

<u>Supporting Information</u>

Discovery of a Novel Aminocyclopropenone Compound that Inhibits BRD4-driven Nucleoporin NUP210 Expression and Attenuates Colorectal Cancer Growth

Hiroya Kondo [1], Kenji Mishiro [2], Yuki Iwashima [3], Qiu Yujia [4], Akiko Kobayashi [2], Keesiang Lim [4], Toshinari Minamoto [5], Kazuma Ogawa [2], Munetaka Kunishima [6], Masaharu Hazawa [2,3,4,*] and Richard W. Wong [2,3,4,*]

[1] Division of Transdisciplinary Sciences, Graduate School of Frontier Science Initiative, Kanazawa University, Kakuma-machi, Kanazawa, Ishikawa 920-1192, Japan; hiro4015@stu.kanazawa-u.ac.jp
[2] Institute for Frontier Science Initiative, Kanazawa University, Kakuma-machi, Kanazawa, Ishikawa 920-1192, Japan; mishiro@p.kanazawa-u.ac.jp (K.M.); akoba@staff.kanazawa-u.ac.jp (A.K.); kogawa@p.kanazawa-u.ac.jp (K.O.)
[3] Laboratory of Molecular Cell Biology, School of Natural System, Institute of Science and Engineering, Kanazawa University, Kakuma-machi, Kanazawa, Ishikawa 920-1192, Japan; yuki090606@stu.kanazawa-u.ac.jp
[4] WPI-Nano Life Science Institute, Kanazawa University, Kakuma-machi, Kanazawa, Ishikawa 920-1192, Japan; porthos0616@gmail.com (Q.Y.); limkeesiang@staff.kanazawa-u.ac.jp (K.L.)
[5] Division of Translational and Clinical Oncology, Cancer Research Institute, Kanazawa University, Kanazawa, Ishikawa 920-0934, Japan; minamoto@staff.kanazawa-u.ac.jp
[6] Faculty of Pharmaceutical Sciences, Institute of Medical, Pharmaceutical, and Health Sciences, Kanazawa University, Kakuma-machi, Kanazawa 920-1192 Japan; kunisima@p.kanazawa-u.ac.jp
* Correspondence: masaharu.akj@gmail.com (M.H.); rwong@staff.kanazawa-u.ac.jp (R.W.); Tel.: +81-076-264-6253

<u>Table of Contents</u>

Synthetic procedure for screened compounds.....S2–S9

^{1}H and ^{13}C NMR spectra of synthesized compounds.....S10–S39

Synthesis of screened compounds

For cyclopropenone **1a**, **1b**, **1f**, **1h**, **1i**, **1j**, **1q**, and **1u** previously synthesized materials were used.[1,2,3]

2-chloro-3-phenylcycloprop-2-en-1-one (**S1**),[1,2] 2-chloro-3-(2,4-dimethoxyphenyl)cycloprop-2-en-1-one (**S2**), [4] and 2-chloro-3-(4-(prop-2-yn-1-yloxy)phenyl)cycloprop-2-en-1-one (**S4**)[3] were synthesized according to the reported procedure. Other commercial reagent and solvents were used as provided without purification.

Synthesis of 2-(dicyclohexylamino)-3-phenylcycloprop-2-en-1-one (**1c**)

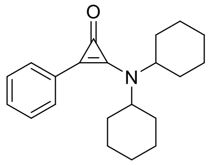

To a stirred solution of 2-chloro-3-phenylcycloprop-2-en-1-one (**S1**) (77% purity, 700 mg, 3.27 mmol) in DCM (5.0 mL), dicyclohexylamine (0.93 mL, 4.67 mmol) and diisopropylethylamine (DIPEA) (1.11 mL, 6.38 mmol) in DCM (5.0 mL) were slowly added at 0 °C. After 30 min, the mixture was diluted in AcOEt (30 mL), washed with 1 M aqueous HCl (40 mL), water (10 mL), and brine (10 mL), dried over Na_2SO_4, filtered, and concentrated *in vacuo*. The residue was purified with SiO_2 column to give a colorless solid. The solid was washed with hexane to give **1c** as a colorless solid (793 mg, 78%).

^{1}H NMR (400 MHz, $CDCl_3$) δ 7.62-7.55 (m, 2H), 7.40-7.30 (m, 3H), 3.80-3.68 (m, 1H), 3.16-3.05 (m, 1H), 2.00-1.55 (m, 14H), 1.50-1.16 (m, 6H). ^{13}C NMR (100 MHz, $CDCl_3$) δ 146.4, 141.3, 128.8, 128.8, 128.6, 125.1, 109.6, 63.4, 56.5, 34.0, 31.6, 25.9, 25.7, 25.0, 24.7. HRMS (DART+) calcd for $C_{21}H_{28}NO$ ([M + H$^+$]$^+$): 310.2171, found: 310.2172.

---

1 Vanos, C. M.; Lambert, T. H., Development of a Catalytic Platform for Nucleophilic Substitution: Cyclopropenone-Catalyzed Chlorodehydration of Alcohols. *Angewandte Chemie International Edition* **2011,** *50* (51), 12222-12226.

2 Mishiro, K.; Yushima, Y.; Kunishima, M., Phototriggered Dehydration Condensation Using an Aminocyclopropenone. *Organic Letters* **2017,** *19* (18), 4912-4915.

3 Mishiro, K.; Yushima, Y.; Kunishima, M., Phototriggered Ketone Formation from an Aminocyclopropenone and a Carboxylic Acid. *The Journal of Organic Chemistry* **2018,** *83* (21), 13595-13603.

4 Mishiro, K.; Nomura, M.; Furuyama, T.; Kunishima, M., Efficiency Enhancement of a Photocatalytic Decarbonylation of an Aminocyclopropenone by Benzothiophene Substitution. *The Journal of Organic Chemistry* **2021,** *86* (4), 3625-3636.

Synthesis of 2-phenyl-3-(pyrrolidin-1-yl)cycloprop-2-en-1-one (**1d**)

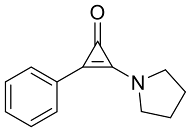

To a stirred solution of **S1** (72% purity, 97 mg, 0.425 mmol) in DCM (1.1 mL), pyrrolidine (83.8 μL, 1.02 mmol) in DCM (1.0 mL) was slowly added at 0 °C. After 1 h, the mixture was diluted in AcOEt (20 mL), washed with 1 M aqueous HCl (10 mL), water (10 mL), and brine (10 mL), dried over Na_2SO_4, filtered, and concentrated *in vacuo*. The residue was washed with AcOEt/hexane (1/3) to give **1d** as a colorless solid (38.5 mg, 45%).

^{1}H NMR (400 MHz, $CDCl_3$) δ 7.60 (d, *J* = 7.3 Hz, 2H), 7.50-7.30 (m, 3H), 3.80 (t, *J* = 6.6 Hz, 2H), 3.69 (t, *J* = 6.4 Hz, 2H), 2.25-1.87 (m, 4H). ^{13}C NMR (100 MHz, $CDCl_3$) δ 145.5, 140.7, 129.0, 128.8, 128.6, 124.3, 111.0, 50.8, 50.6, 25.6, 25.5. HRMS (DART+) calcd for $C_{13}H_{14}NO$ ([M + H$^+$]$^+$): 200.1075, found: 200.1084.

Synthesis of 2-phenyl-3-(2,2,6,6-tetramethylpiperidin-1-yl)cycloprop-2-en-1-one (**1e**)

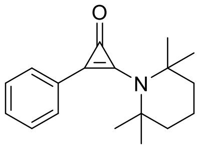

To a stirred solution of **S1** (77% purity, 86 mg, 0.4 mmol) in DCM (0.4 mL), 2,2,6,6-tetramethylpiperidine (0.204 mL, 1.2 mmol) in DCM (0.4 mL) was slowly added at 0 °C. After 30 min, the mixture was diluted in AcOEt, washed with 1 M aqueous HCl, water, and brine, dried over Na_2SO_4, filtered, and concentrated *in vacuo*. The residue was purified with SiO_2 column to give a pale yellow solid. The solid was washed with Et_2O/hexane (1/3) to give **1e** as a colorless solid (72.1 mg, 67%).

^{1}H NMR (400 MHz, $CDCl_3$) δ 7.68-7.59 (m, 2H), 7.45-7.32 (m, 3H), 1.78-1.60 (m, 6H), 1.490 (s, 6H), 1.486 (s, 6H). ^{13}C NMR (100 MHz, $CDCl_3$) δ 149.1, 142.2, 129.1, 128.8, 128.6, 126.5, 113.6, 57.7, 39.5, 29.8, 16.2. HRMS (DART+) calcd for $C_{18}H_{24}NO$ ([M + H$^+$]$^+$): 270.1858, found: 270.1860.

Synthesis of 2-phenyl-3-(phenylamino)cycloprop-2-en-1-one (**1g**)

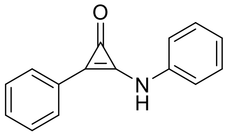

To a stirred solution of **S1** (72% purity, 100 mg, 0.44 mmol) in DCM (1.2 mL), aniline (120 μL,

1.31 mmol) in DCM (1.0 mL) was slowly added at 0 °C. A colorless solid gradually precipitated in the reaction mixture. After 1 h, to the mixture, AcOEt/hexane (2.0 mL/6.0 mL) was added. The solid was collected by filtration, washed with AcOEt/hexane (1/3) and 1 M aqueous HCl, and dried *in vacuo* to give **1g** as a colorless solid (73.9 mg, 76%).
^{1}H NMR (400 MHz, DMSO-d6) δ 11.0 (s, 1H), 7.81 (d, *J* = 6.9 Hz, 2H), 7.57 (t, *J* = 7.3 Hz, 2H), 7.50 (t, *J* = 7.1 Hz, 1H), 7.43 (t, *J* = 7.8 Hz, 2H), 7.32 (d, *J* = 7.8 Hz, 2H), 7.09 (t, *J* = 7.1 Hz, 1H). ^{13}C NMR (100 MHz, DMSO-d6) δ 143.1, 140.5, 137.9, 130.1, 129.8, 129.5, 129.2, 123.7, 122.9, 115.6, 112.7. HRMS (DART+) calcd for $C_{15}H_{12}NO$ ($[M + H^+]^+$): 222.0919, found: 222.0911.

Synthesis of *N*-methyl-*N*-(3-oxo-2-phenylcycloprop-1-en-1-yl)methanesulfonamide (**1k**)

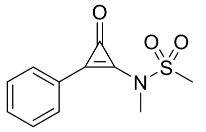

To 40% methylamine in MeOH (40%, 2.2 mL, 25.8 mmol), methanesulfonyl chloride (0.5 mL, 6.46 mmol) was slowly added at 0 °C. After 20 min, the mixture was warmed to rt, concentrated *in vacuo*. The residue was suspended in DCM (10 mL), dried over Na_2SO_4, filtered, and concentrated *in vacuo* to give *N*-methylmethanesulfonamide as a colorless oil (523 mg, 74%). The oil was used in the following reaction without further purification. To a stirred mixture of NaH (60% oil dispersion) (31 mg, 0.77 mmol) in THF (2.0 mL), *N*-methylmethanesulfonamide (92 mg, 0.84 mmol) was added at 0 °C. After 30 min, to the stirred mixture, a solution of **S1** (77% purity, 150 mg, 0.702 mmol) in THF (1.5 mL) was slowly added at 0 °C. After 60 min, the reaction was quenched with 1 M HCl (1.0 mL), extracted with DCM (20 mL) dried over Na_2SO_4, filtered, and concentrated *in vacuo*. The residue was purified with SiO_2 column (AcOEt/Hexane = 1/1–1/0) to give **1k** as a colorless solid (62 mg, 37%).
^{1}H NMR (400 MHz, $CDCl_3$) δ 7.83-7.77 (m, 2H), 7.54-7.42 (m, 3H), 3.65 (s, 3H), 3.28 (s, 3H). ^{13}C NMR (100 MHz, $CDCl_3$) δ 145.4, 138.1, 131.4, 130.4, 129.2, 122.8, 122.6, 40.2, 37.3. HRMS (DART+) calcd for $C_{11}H_{12}NO_3S$ ($[M + H^+]^+$): 238.0538, found: 238.0548.

Synthesis of *N*-isopropyl-*N*-(3-oxo-2-phenylcycloprop-1-en-1-yl)methanesulfonamide (**1l**)

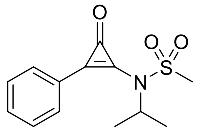

To a stirred solution of isopropylamine (0.508 mL, 6.2 mmol) in DCM (3.0 mL), methanesulfonyl chloride (0.2 mL, 2.58 mmol) was slowly added at 0 °C. After 20 min, the mixture was diluted with DCM (20 mL), washed with 0.1 M aqueous HCl, water, and brine, dried over Na_2SO_4, filtered, and concentrated *in vacuo* to give *N*-isopropylmethanesulfonamide as a colorless solid (223 mg,

63%). The solid was used in the following reaction without further purification. To a stirred mixture of NaH (60% oil dispersion) (23.2 mg, 0.58 mmol) in THF (2.0 mL), *N*-isopropylmethanesulfonamide (53 mg, 0.386 mmol) was added at 0 °C. After 30 min, to the stirred mixture, a solution of **S1** (72% purity, 97 mg, 0.425 mmol) in THF (1.0 mL) was slowly added at 0 °C. After 30 min, the reaction was quenched with 1 M HCl (1.0 mL), extracted with AcOEt (20 mL), washed with water (10 mL) and brine (10 mL), dried over Na_2SO_4, filtered, and concentrated *in vacuo*. The residue was purified with SiO_2 column (AcOEt/Hexane = 1/3–1/1) to give **1l** as a colorless solid (74.6 mg, 73%).

^{1}H NMR (400 MHz, $CDCl_3$) δ 8.10-7.87 (m, 2H), 7.60-7.40 (m, 3H), 4.52 (sep, *J* = 6.6 Hz, 1H), 3.21 (s, 3H), 1.51 (d, *J* = 6.4 Hz, 6H). ^{13}C NMR (100 MHz, $CDCl_3$) δ 145.8, 134.8, 131.2, 130.9, 128.9, 123.2, 123.0, 53.7, 41.8, 22.3. HRMS (DART+) calcd for $C_{13}H_{16}NO_3S$ ([M + H$^+$]$^+$): 266.0851, found: 266.0842.

Synthesis of *N*-(tert-butyl)-*N*-(3-oxo-2-phenylcycloprop-1-en-1-yl)methanesulfonamide (**1m**)

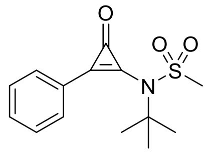

To a stirred solution of *tert*-butylamine (0.2 mL, 1.9 mmol) in DCM (8.5 mL) and DIPEA (0.45 mL, 2.6 mL), methanesulfonyl chloride (0.134 mL, 1.73 mmol) was slowly added at 0 °C. After 10 min, the mixture was warmed to rt and concentrated in vacuo. The residue was dissolved in AcOEt, washed with 0.1 M HCl, water, and brine, dried over Na_2SO_4, filtered, and concentrated *in vacuo* to give *N*-(*tert*-butyl)methanesulfonamide as a colorless oil (130 mg, 45%). The oil was used in the following reaction without further purification. To a stirred mixture of NaH (60% oil dispersion) (34 mg, 0.852 mmol) in THF (1.0 mL), *N*-(*tert*-butyl)methanesulfonamide (103 mg, 0.681 mmol) was added at 0 °C. After 30 min, to the stirred mixture, a solution of **S1** (77% purity, 94 mg, 0.568 mmol) in THF (1.0 mL) was slowly added at 0 °C. After 2.5 h, the reaction was quenched with 1 M HCl (1.0 mL), extracted with DCM (20 mL) dried over Na_2SO_4, filtered, and concentrated *in vacuo*. The residue was purified with SiO_2 column (AcOEt/Hexane = 1/2–1/1) to give **1m** as a colorless solid (32.3 mg, 20%).

^{1}H NMR (400 MHz, $CDCl_3$) δ 7.99-7.92 (m, 2H), 7.53-7.42 (m, 3H), 3.26 (s, 3H), 1.73 (s, 9H). ^{13}C NMR (100 MHz, $CDCl_3$) δ 146.2, 137.7, 131.15, 131.10, 128.9, 123.4, 123.1, 66.7, 43.8, 30.3. HRMS (DART+) calcd for $C_{14}H_{18}NO_3S$ ([M + H$^+$]$^+$): 280.1007, found: 280.1009.

Synthesis of 2-(diethylamino)-3-(2,4-dimethoxyphenyl)cycloprop-2-en-1-one (**1n**)

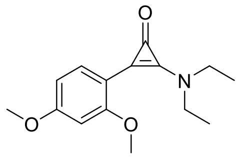

To a stirred solution of 2-chloro-3-(2,4-dimethoxyphenyl)cycloprop-2-en-1-one (**S2**) (40 mg, 0.178 mmol) in DCM (0.6 mL), a solution of diethylamine (44.2 μL, 0.427 mmol) in DCM (1.2 mL) was added at 0 °C under N_2 atmosphere. After 1 h, the mixture was diluted with AcOEt, washed with 0.1 N HCl, H_2O, and brine, dried over Na_2SO_4, filtered, and concentrated *in vacuo*. The residue was purified by SiO_2 column (eluent: AcOEt:hexane = 1:2 – 1:0) to give a colorless solid. The solid was washed with hexane to give **1n** as a colorless solid (25.5 mg, 55%).

^{1}H NMR (400 MHz, $CDCl_3$) δ 7.52 (d, *J* = 8.3 Hz, 1H), 6.51 (d, *J* = 8.3 Hz, 1H), 6.47 (s, 1H), 3.87 (s, 3H), 3.83 (s, 3H), 3.60 (q, *J* = 6.4 Hz, 2H), 3.43 (q, *J* = 6.4 Hz, 2H), 1.36 (t, *J* = 6.4 Hz, 3H), 1.26 (t, *J* = 6.4 Hz, 3H). ^{13}C NMR (100 MHz, $CDCl_3$) δ 161.7, 157.9, 146.3, 139.6, 132.6, 107.69, 107.66, 104.8, 98.4, 55.4, 55.2, 46.9, 45.1, 14.3, 13.8. HRMS (DART+) calcd for $C_{15}H_{20}NO_3$ ([M + H$^+$]$^+$): 262.1443, found: 262.1435.

Synthesis of 2-chloro-3-(2,4,6-trimethoxyphenyl)cycloprop-2-en-1-one (**S3**)

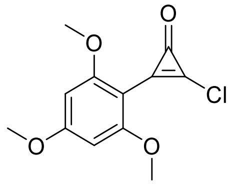

To a stirred solution of $AlCl_3$ (149 mg, 1.1 mmol) in $MeNO_2$ (0.4 mL), tetrachlorocyclopropene (0.10 mL, 0.821 mmol) was added at 0 °C under N_2 atmosphere. After 30 min, to the mixture, DCM (1.6 mL) was added and the mixture was cooled to –78 °C. To the mixture, a solution of 1,3,5-trimethoxybenzene (125 mg, 0.746 mmol) in DCM (2.0 mL) was added. After stirring for 20 min at −78 °C, the mixture was diluted with DCM (10 mL) and quenched with ice cooled water (5 mL). The mixture was warmed to 0 °C and stirred for 30 min. Then the DCM phase was separated and aqueous phase was extracted with DCM (5 mL × 2). Combined DCM phase was dried over Na_2SO_4, filtered and concentrated *in vacuo*. The residue was purified by SiO_2 column (eluent: AcOEt:hexane = 1:3 – 1:1) to give **S3** as a colorless solid (34.5 mg, 18%).

^{1}H NMR (400 MHz, $CDCl_3$) δ 6.08 (s, 2H), 3.95 (s, 6H), 3.89 (s, 3H). ^{13}C NMR (100 MHz, $CDCl_3$) δ 167.2, 162.7, 149.8, 149.5, 125.0, 95.7, 90.0, 56.1, 55.7. HRMS (DART+) calcd for $C_{12}H_{12}ClO_4$ ([M + H$^+$]$^+$): 255.0424, found: 255.0420.

Synthesis of 2-(diisopropylamino)-3-(2,4,6-trimethoxyphenyl)cycloprop-2-en-1-one (**1o**)

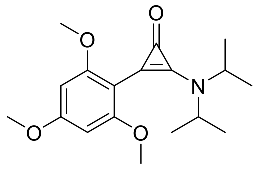

To a stirred solution of **S3** (20 mg, 0.0785 mmol) in DCM (0.4 mL), a solution of diisopropylamine (26.6 μL, 0.189 mmol) in DCM (0.4 mL) was added at 0 °C under N_2 atmosphere. After 1 h, the mixture was diluted with AcOEt, washed with 1 M HCl, H_2O, and brine, dried over Na_2SO_4, filtered, and concentrated *in vacuo*. The residue was purified by SiO_2 column (eluent: AcOEt:hexane = 1:1 – 1:0, then AcOEt:acetone = 1:1) to give a colorless solid. The solid was washed with hexane to give **1o** as a colorless solid (19.2 mg, 67%).

^{1}H NMR (400 MHz, $CDCl_3$) δ 6.12 (s, 2H), 4.48 (sep, *J* = 6.6 Hz, 1H), 3.88 (s, 6H), 3.84 (s, 3H), 3.54 (sep, *J* = 6.4 Hz, 1H), 1.36 (d, *J* = 6.4 Hz, 6H), 1.24 (d, *J* = 6.9 Hz, 6H). ^{13}C NMR (100 MHz, $CDCl_3$) δ 162.2, 159.6, 146.8, 140.2, 103.9, 97.7, 90.2, 55.8, 55.4, 51.6, 46.0, 23.7, 21.0. HRMS (DART+) calcd for $C_{18}H_{26}NO_4$ ([M + H$^+$]$^+$): 320.1862, found: 320.1864.

Synthesis of 2-(diethylamino)-3-(4-(prop-2-yn-1-yloxy)phenyl)cycloprop-2-en-1-one (**1p**)

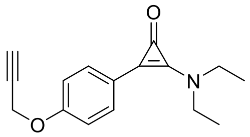

To a stirred solution of 2-chloro-3-(4-(prop-2-yn-1-yloxy)phenyl)cycloprop-2-en-1-one (**S4**) (30 mg, 0.137 mmol) in DCM (1.3 mmol), diethylamine (34 μL, 0.329 mmol) was added at 0 °C. After 10 min, the mixture was diluted in AcOEt, washed with H_2O, dried over Na_2SO_4, filtered and concentrated *in vacuo* to give **1p** as a colorless solid (28.4 mg, 81%).

^{1}H NMR (400 MHz, $CDCl_3$) δ 7.52 (d, *J* = 8.2 Hz, 2H), 7.02 (d, *J* = 8.7 Hz, 2H), 4.72 (d, *J* = 1.8 Hz, 2H), 3.59 (q, *J* = 6.9 Hz, 2H), 3.44 (q, *J* = 6.9 Hz, 2H), 2.56 (t, *J* = 1.8 Hz, 1H), 1.47-1.25 (m, 6H). ^{13}C NMR (100 MHz, $CDCl_3$) δ 157.9, 145.4, 140.9, 130.5, 118.4, 115.3, 110.6, 77.9, 75.9, 55.8, 47.4, 46.9, 14.3. HRMS (DART+) calcd for $C_{16}H_{18}NO_2$ ([M + H$^+$]$^+$): 256.1338, found: 256.1328.

Synthesis of 2-(methyl(phenyl)amino)-3-(4-(prop-2-yn-1-yloxy)phenyl)cycloprop-2-en-1-one (**1q**)

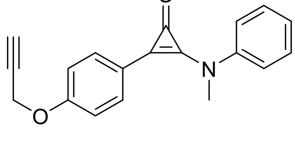

To a stirred solution of **S4** (23 mg, 0.105 mmol) in DCM (0.4 mmol), N-methylaniline (27 μL, 0.25 mmol) was added at 0 °C. After 10 min, the mixture was directly purified with SiO_2 column

(AcOEt/hexane = 1/2–1/0) to give **1q** as a colorless solid (25.5 mg, 84%).
^{1}H NMR (400 MHz, $CDCl_3$) δ 7.70 (d, *J* = 8.2 Hz, 2H), 7.58-6.85 (m, 5H), 7.05 (*d*, J = 8.2 Hz, 2H), 4.74 (br s, 2H), 3.73 (s, 3H), 2.57 (br s, 1H). ^{13}C NMR (100 MHz, $CDCl_3$) δ 158.7, 145.0, 142.6, 139.3, 132.6, 131.3, 129.7, 129.4, 123.7, 121.5, 117.4, 116.1, 115.5, 115.0, 77.8, 76.1, 55.8, 38.5. HRMS (DART+) calcd for $C_{19}H_{16}NO_2$ ($[M + H^+]^+$): 290.1181, found: 290.1188.

Synthesis of 2-(diethylamino)-3-(2,3,4,5,6-pentamethylphenyl)cycloprop-2-en-1-one (**1s**)

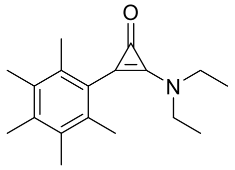

To a stirred solution of $AlCl_3$ (2.09 g, 15.6 mmol) in $MeNO_2$/DCM (3.9 mL/7.8 mL), tetrachlorocyclopropene (0.5 mL, 4.1 mmol) was added at 0 °C under N_2 atmosphere. After stirring for 10 min, the mixture was cooled to –78 °C. To the mixture, a solution of pentamethylbenzene (580 mg, 3.91 mmol) in DCM (27 mL) was added over 5 min. After stirring for 10 min at –78°C, the mixture was poured into ice cold water. DCM phase was separated and the aqueous phase was extracted with DCM (10 mL × 2). Combined DCM phase was dried over Na_2SO_4, filtered and concentrated *in vacuo*. The residue was dissolved in acetone/water (20 mL/5 mL) at 0 °C and stirred for 60 min. After completion of the hydrolysis, the mixture was extracted with DCM (10 mL × 3). The combined DCM phases was cooled to 0 °C. To the DCM solution, diethylamine (1.21 mL, 11.7 mmol) was added at 0 °C. After 10 min, the mixture was concentrated *in vacuo*. The residue was purified by SiO_2 column chromatography (eluent: AcOEt/ hexane = 1/1 – 1/0) to give **1s** as a pale yellow solid (557 mg, 52% over 3 steps).
^{1}H NMR (400 MHz, $CDCl_3$) δ 3.39 (brs, 4H), 2.27 (s, 6H), 2.24 (s, 3H), 2.21 (s, 3H), 1.40 (brs, 3H), 1.16 (s, 3H). ^{13}C NMR (100 MHz, $CDCl_3$) δ 147.1, 146.0, 135.7, 132.7, 132.2, 124.3, 113.8, 45.4, 18.6, 16.8, 16.4, 13.8. HRMS (DART+) calcd for $C_{18}H_{26}NO$ ($[M + H^+]^+$): 272.2014, found: 272.2010.

Synthesis of 2-(diethylamino)-3-(thiophen-2-yl)cycloprop-2-en-1-one (**1t**)

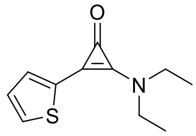

To a stirred solution of $AlCl_3$ (875 mg, 6.56 mmol) in $MeNO_2$ (0.8 mL), tetrachlorocyclopropene (0.2 mL, 1.64 mmol) was added at 0 °C under N_2 atmosphere. After stirring for 20 min, to the mixture DCM (1.6 mL) was added and the mixture was cooled to –78 °C. To the mixture, a solution of thiophene (119 μL, 1.49 mmol) in DCM (1.6 mL) was added over 10 min. After stirring for 30 min at –78°C, the mixture was diluted with DCM (10 mL) and poured into ice

cold water. DCM phase was separated and the aqueous phase was extracted with DCM (5 mL × 2). Combined DCM phase was dried over Na_2SO_4, filtered and concentrated *in vacuo*. The residue was dissolved in acetone/water (4.0 mL/1.0 mL) at 0 °C and stirred for 30 min. After completion of the hydrolysis, a solution of diethylamine (0.93 mL, 8.99 mmol) in DCM (10 mL) was added at 0 °C. After 30 min, the DCM phase was separated, and the aqueous phase was extracted with DCM (5.0 mL × 2). The DCM phases were combined, dried over Na_2SO_4, filtered and concentrated *in vacuo*. The residue was purified by SiO_2 column chromatography (eluent: AcOEt/ hexane = 1/1 – 1/0). The purified material was washed with hexane to give **1t** as a pale yellow solid (125.7 mg, 41% over 3 steps).
^{1}H NMR (400 MHz, $CDCl_3$) δ 7.39 (d, *J* = 3.7 Hz, 1H), 7.37 (d, *J* = 5.0 Hz, 1H), 7.18-7.02 (m, 1H), 3.61 (q, *J* = 7.3 Hz, 2H), 3.42 (q, *J* = 7.3 Hz, 2H), 1.38 (t, *J* = 7.3 Hz, 3H), 1.34 (t, *J* = 7.3 Hz, 3H). ^{13}C NMR (100 MHz, $CDCl_3$) δ 142.7, 138.4, 130.1, 128.1, 127.4, 125.6, 104.5, 46.9, 46.7, 14.5, 14.2. HRMS (DART+) calcd for $C_{11}H_{14}NOS$ ([M + H$^+$]$^+$): 208.0796, found: 208.0798.

Synthesis of *N,N*-diethyl-2,4-dimethoxybenzamide (**2n**)

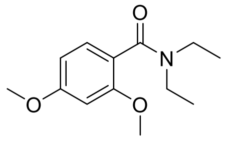

To a stirred mixture of 2,4-dimethoxybenzoic acid (182 mg, 1.0 mmol) in DCM (1.0 mL), oxalyl chloride (93 μL, 1.1 mmol) and DMF (1 drop) were added at rt. After 1 h, the mixture was cooled to 0 °C. Then diethylamine (0.41 mL, 4.0 mmol) was added to the mixture at 0 °C. After 1 h, the mixture was diluted with AcOEt, washed with saturated aqueous $NaHCO_3$, dried over Na_2SO_4, filtered, and concentrated *in vacuo*. The residue was purified with SiO_2 column (AcOEt/hexane = 1/2–1/1) to give **2n** as a colorless oil (141 mg, 59%).
^{1}H NMR (400 MHz, $CDCl_3$) δ 7.12 (d, *J* = 8.2 Hz, 1H), 6.49 (dd, *J* = 8.2, 2.3 Hz, 1H), 6.45 (d, 2.3 Hz, 1H), 3.81 (s, 3H), 3.79 (s, 3H), 3.55 (brs, 2H), 3.16 (q, *J* = 7.3 Hz, 2H), 1.23 (t, *J* = 7.3 Hz, 3H), 1.03 (t, *J* = 7.3 Hz, 3H). ^{13}C NMR (100 MHz, $CDCl_3$) δ 168.7, 161.1, 156.5, 128.2, 119.7, 104.5, 98.5, 55.4, 55.3, 42.8, 38.8, 13.9, 12.8. HRMS (DART+) calcd for $C_{13}H_{20}NO_3$ ([M + H$^+$]$^+$): 238.1443, found: 238.1455.

^{1}H NMR spectrum of **1c** (400 MHz, $CDCl_3$)

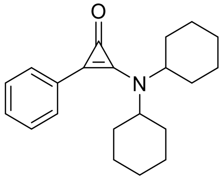

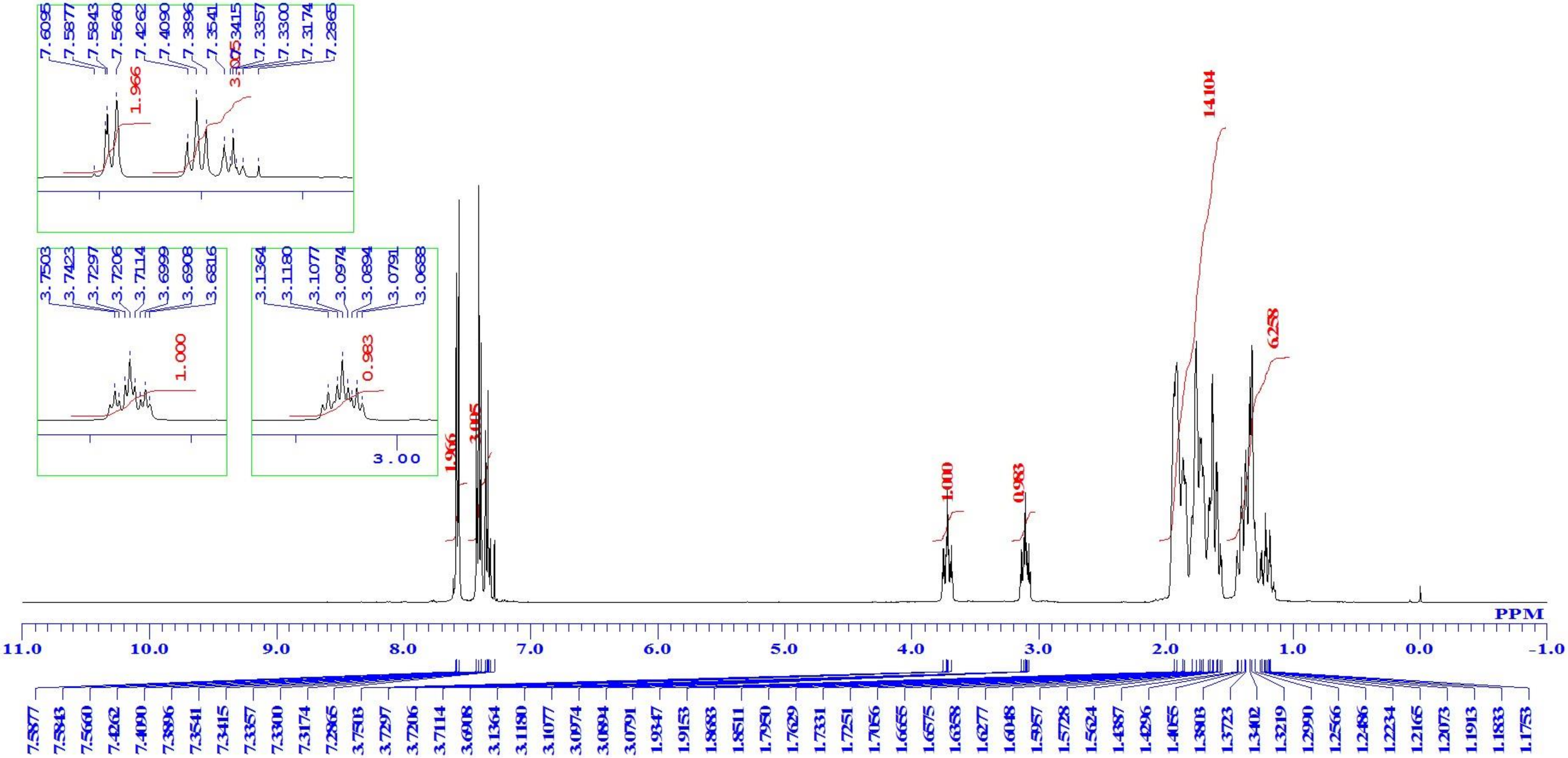

^{13}C NMR spectrum of **1c** (100 MHz, $CDCl_3$)

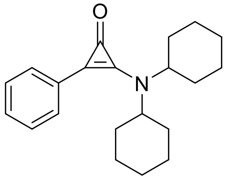

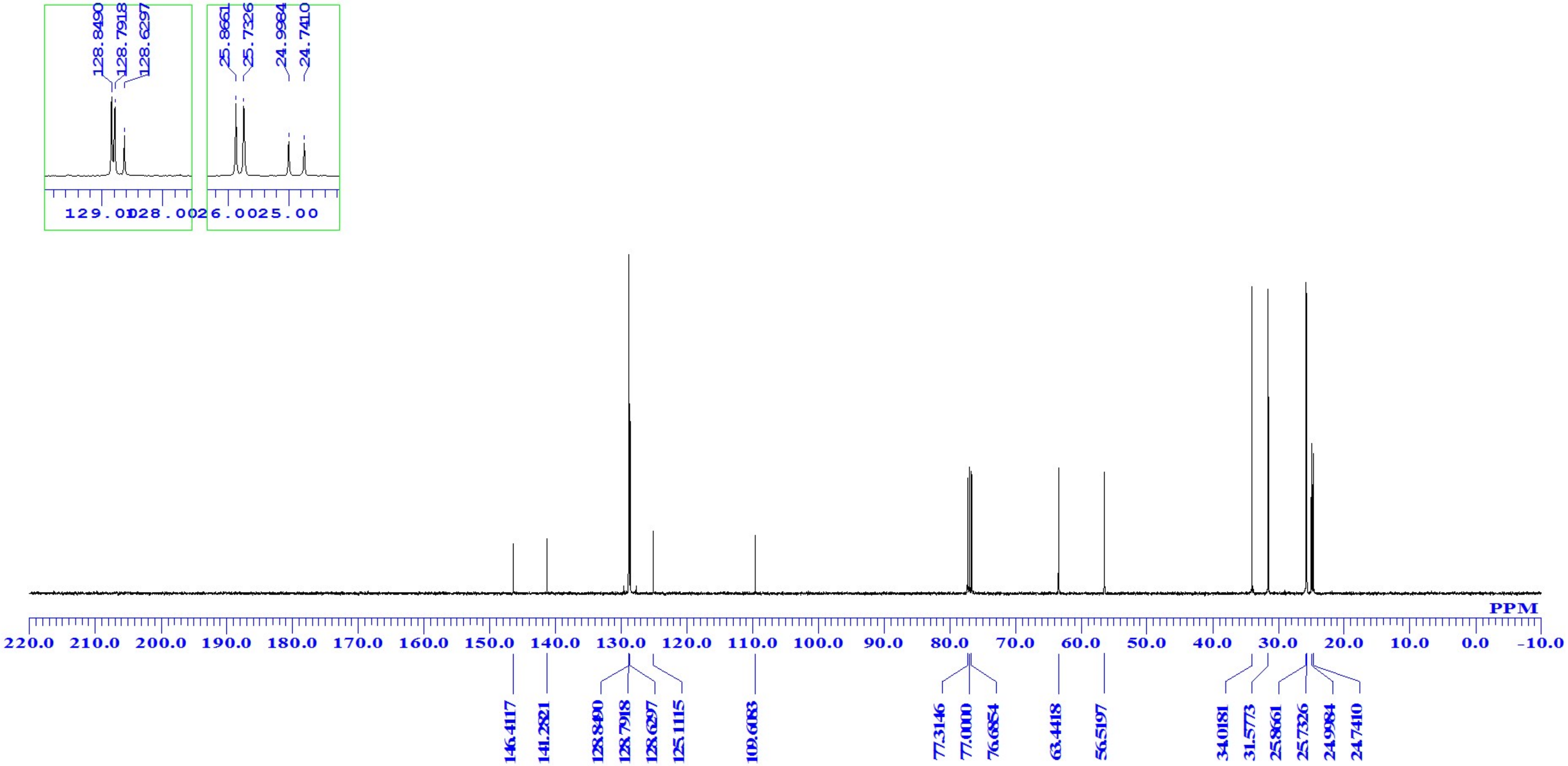

^{1}H NMR spectrum of **1d** (400 MHz, $CDCl_3$)

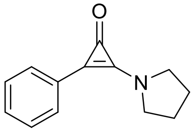

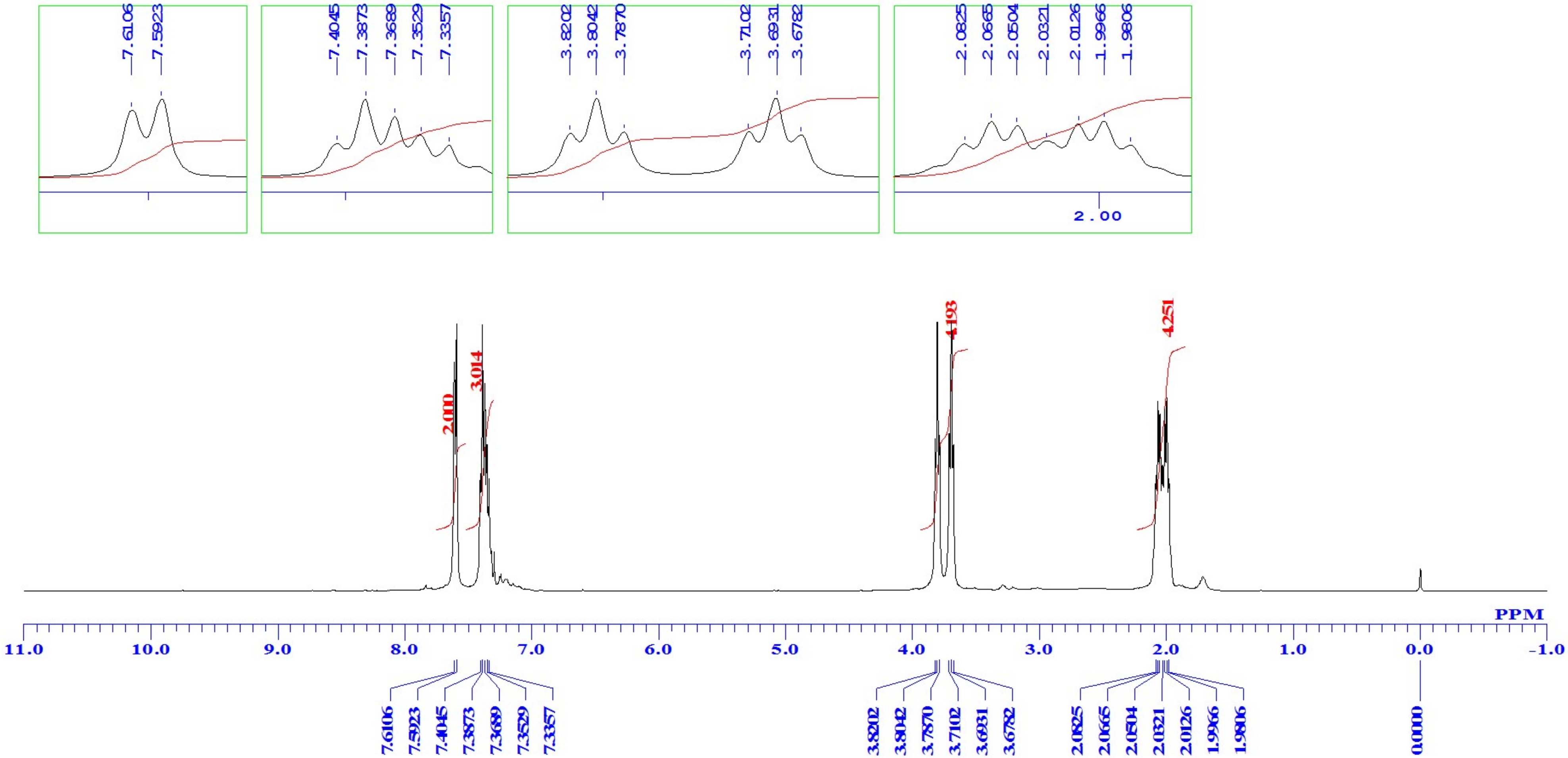

^{13}C NMR spectrum of **1d** (100 MHz, $CDCl_3$)

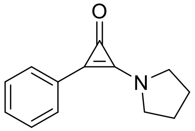

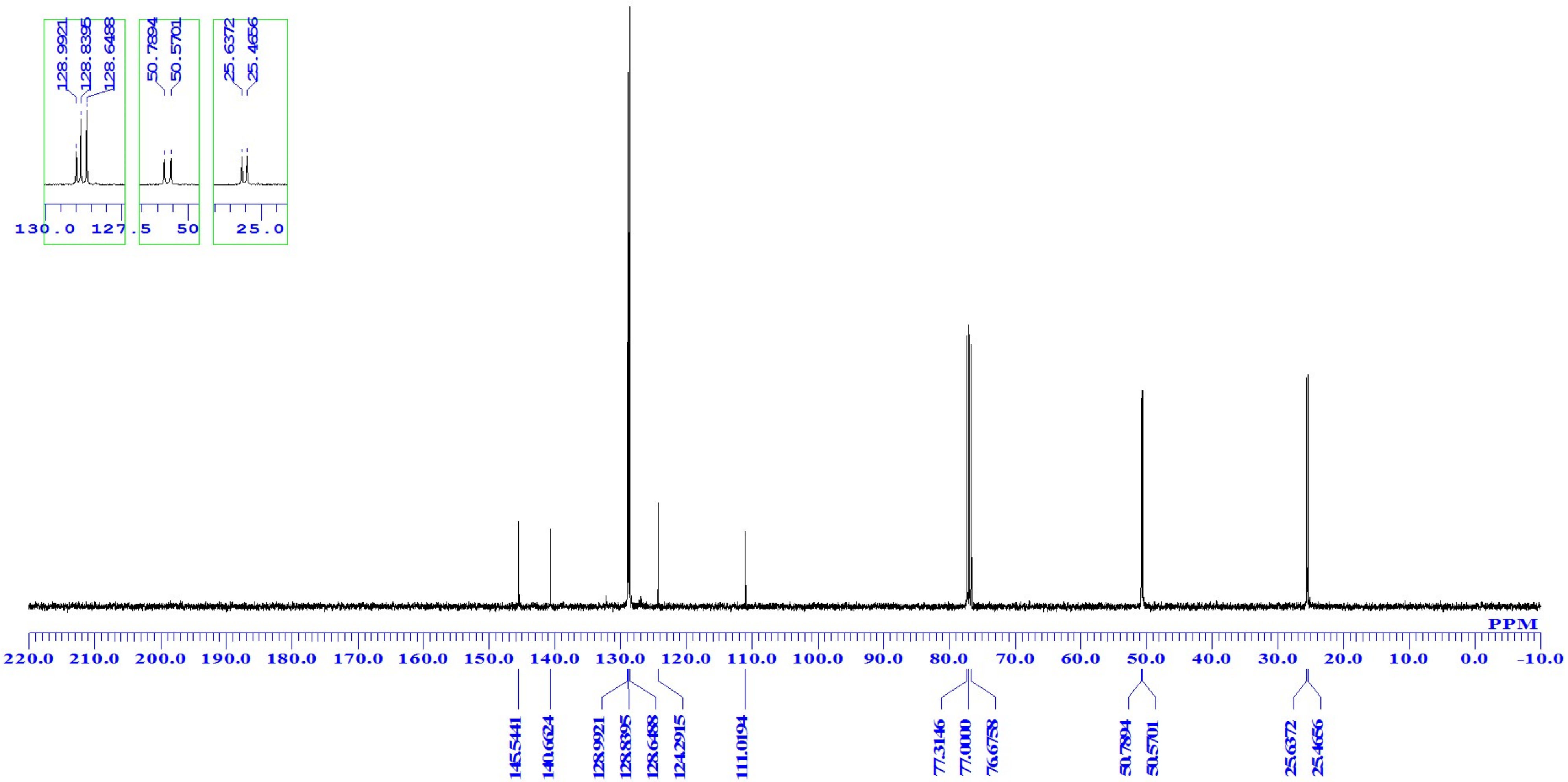

^{1}H NMR spectrum of **1e** (400 MHz, $CDCl_3$)

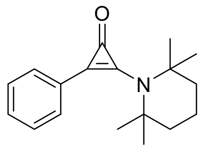

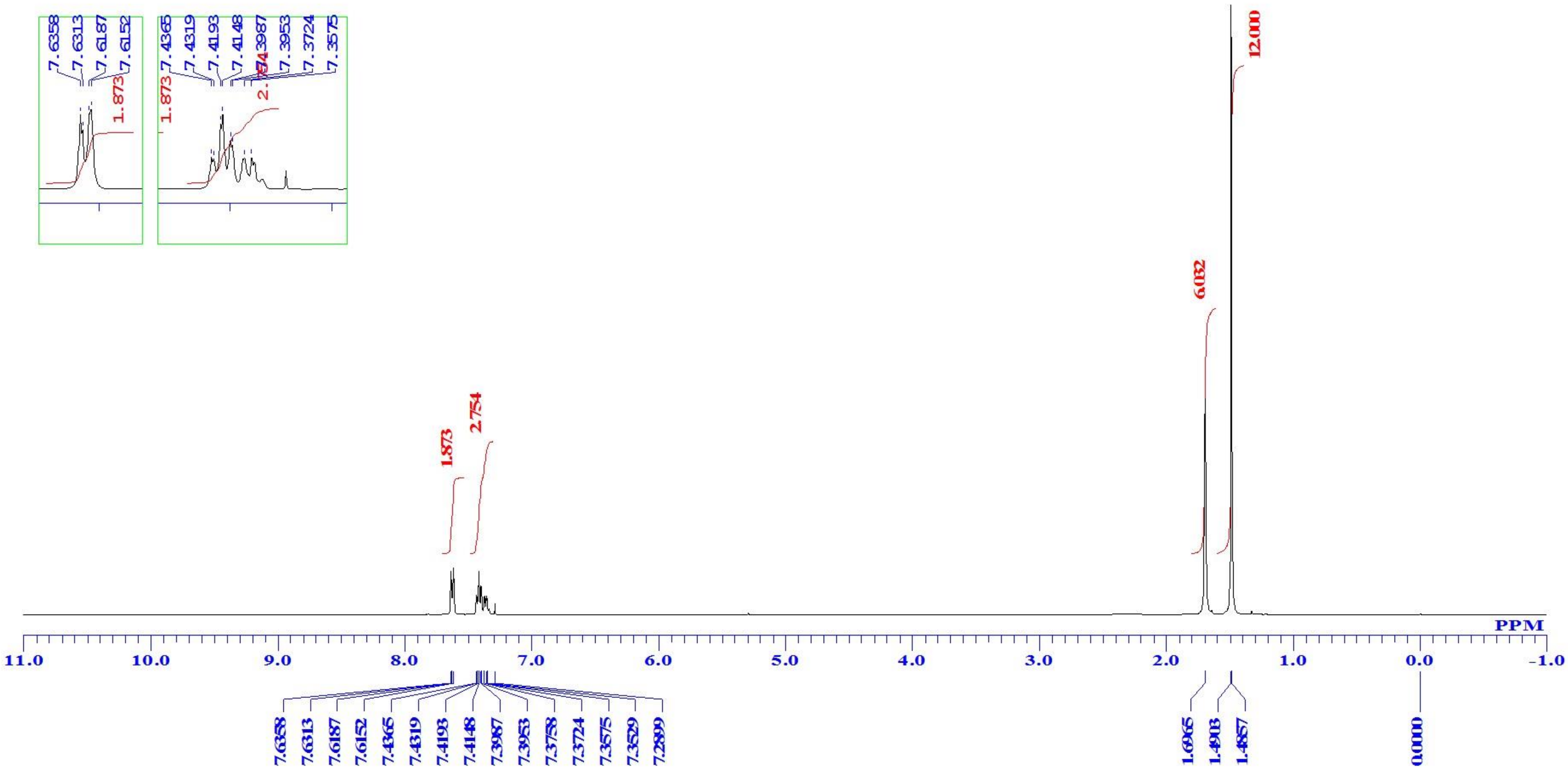

^{13}C NMR spectrum of **1e** (100 MHz, $CDCl_3$)

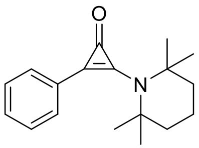

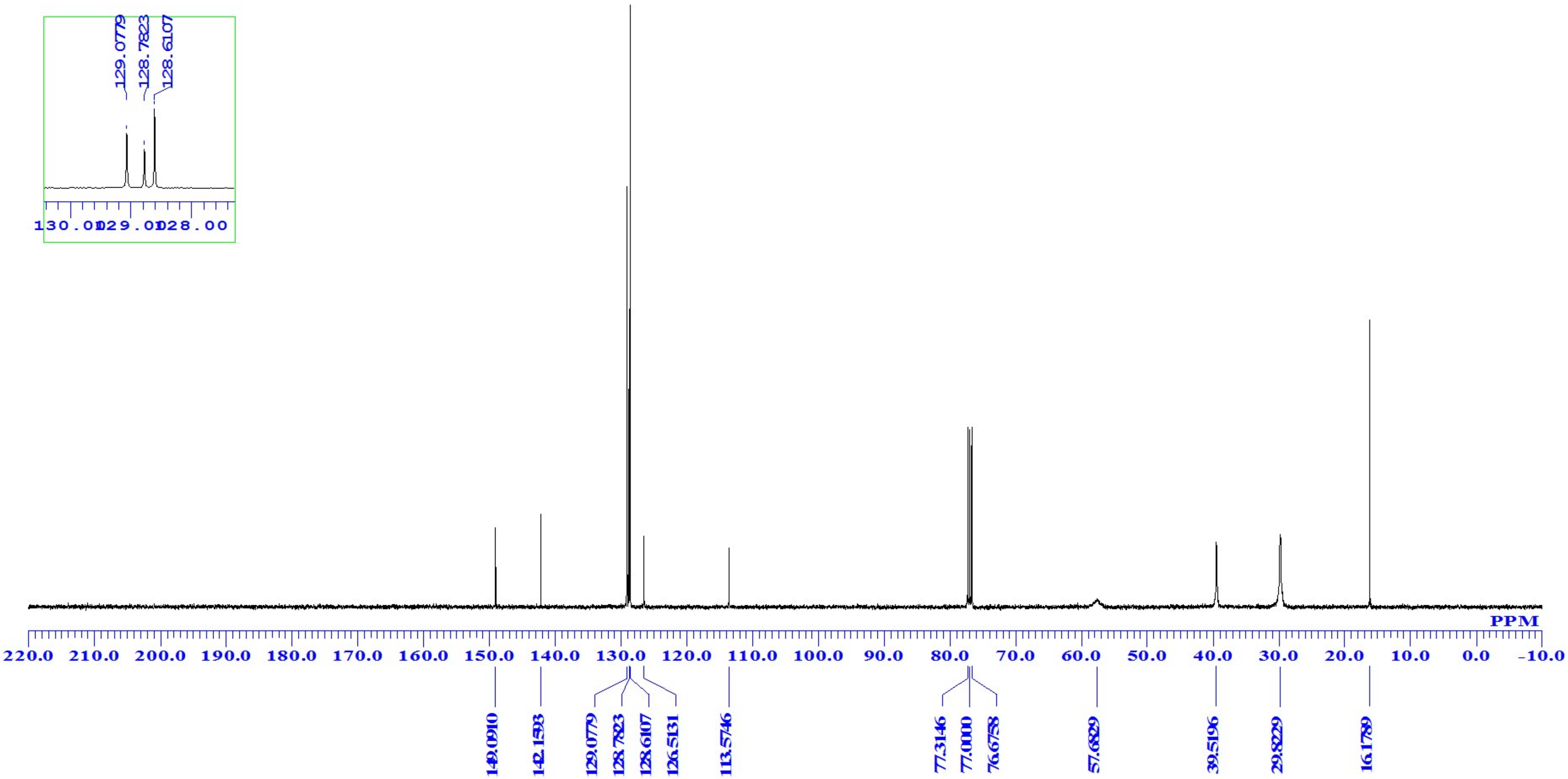

^{1}H NMR spectrum of **1g** (400 MHz, DMSO-d6)

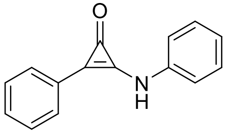

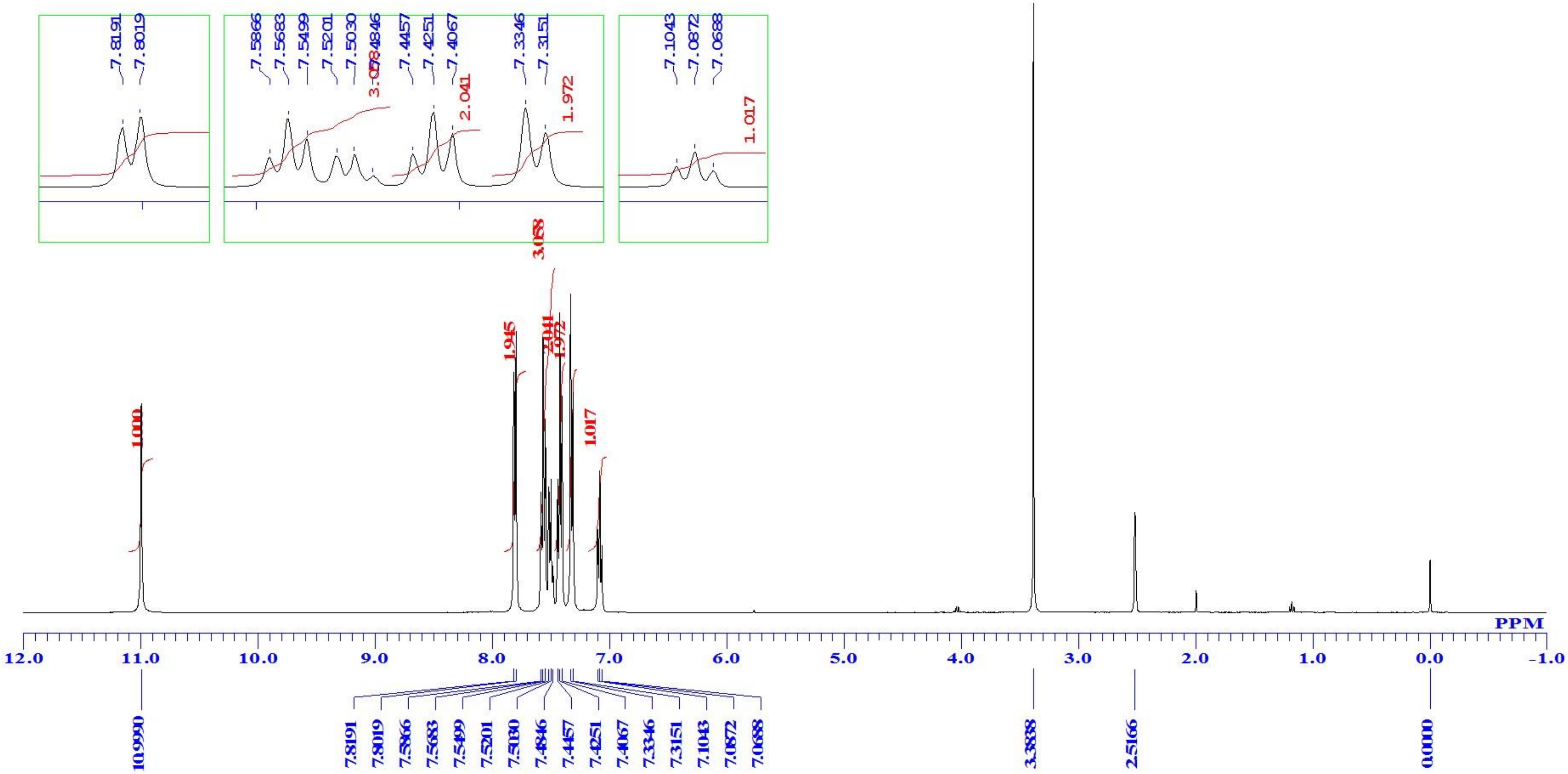

^{13}C NMR spectrum of **1g** (100 MHz, DMSO-d6)

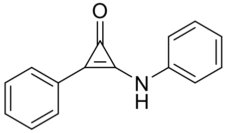

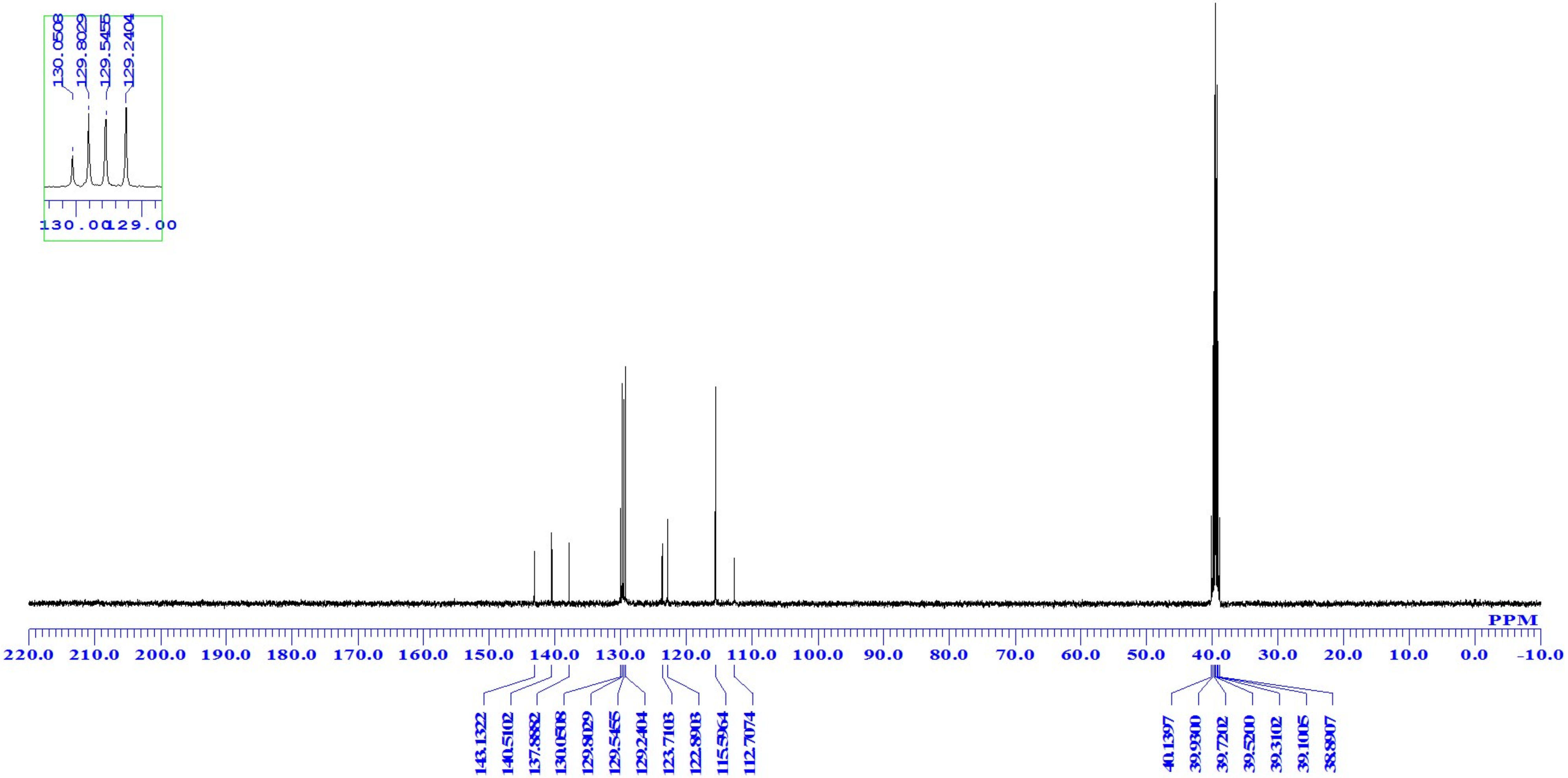

^{1}H NMR spectrum of **1k** (400 MHz, $CDCl_3$)

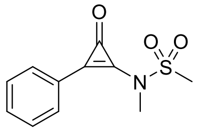

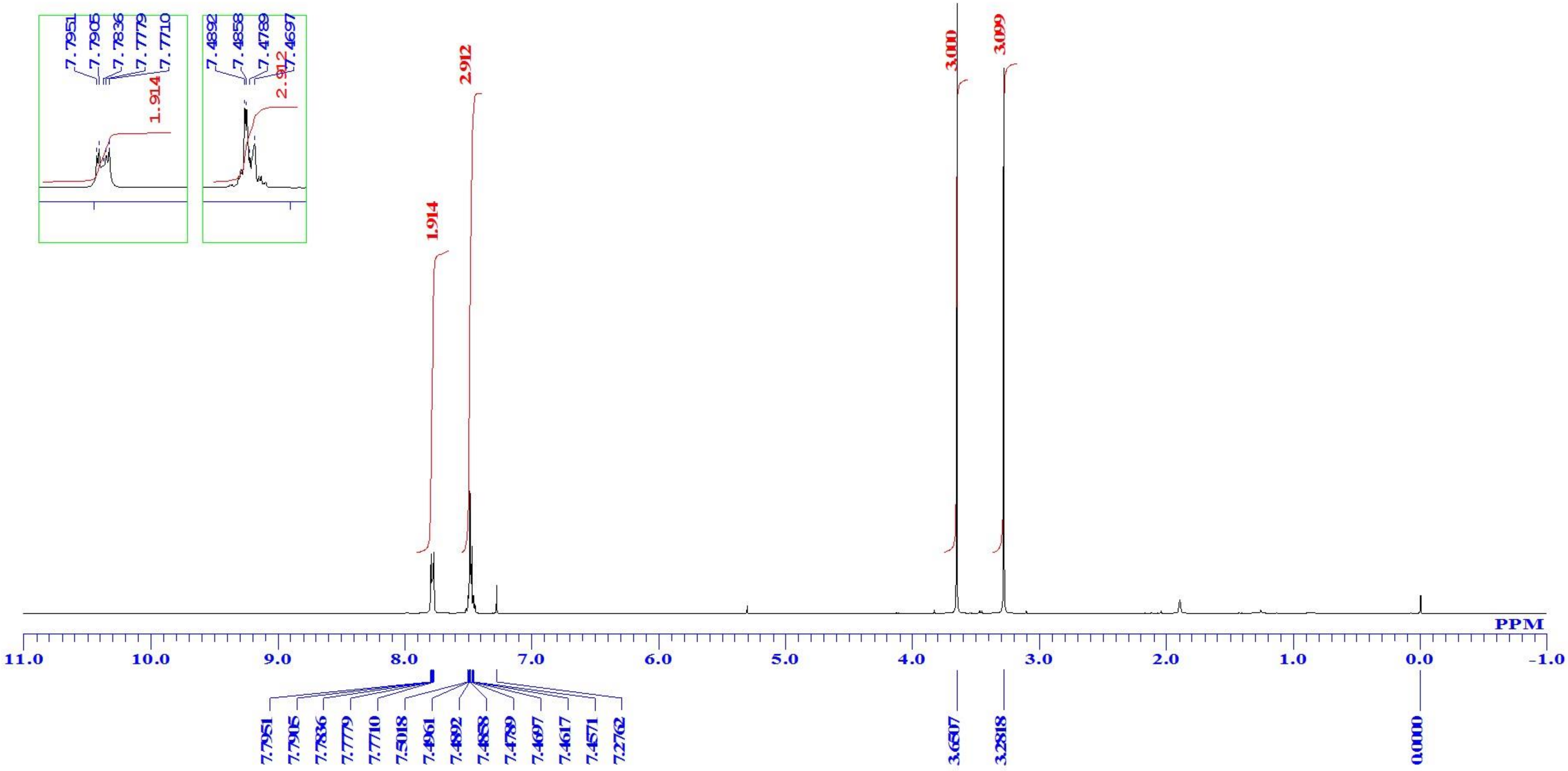

^{13}C NMR spectrum of **1k** (100 MHz, CDCl$_3$)

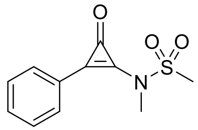

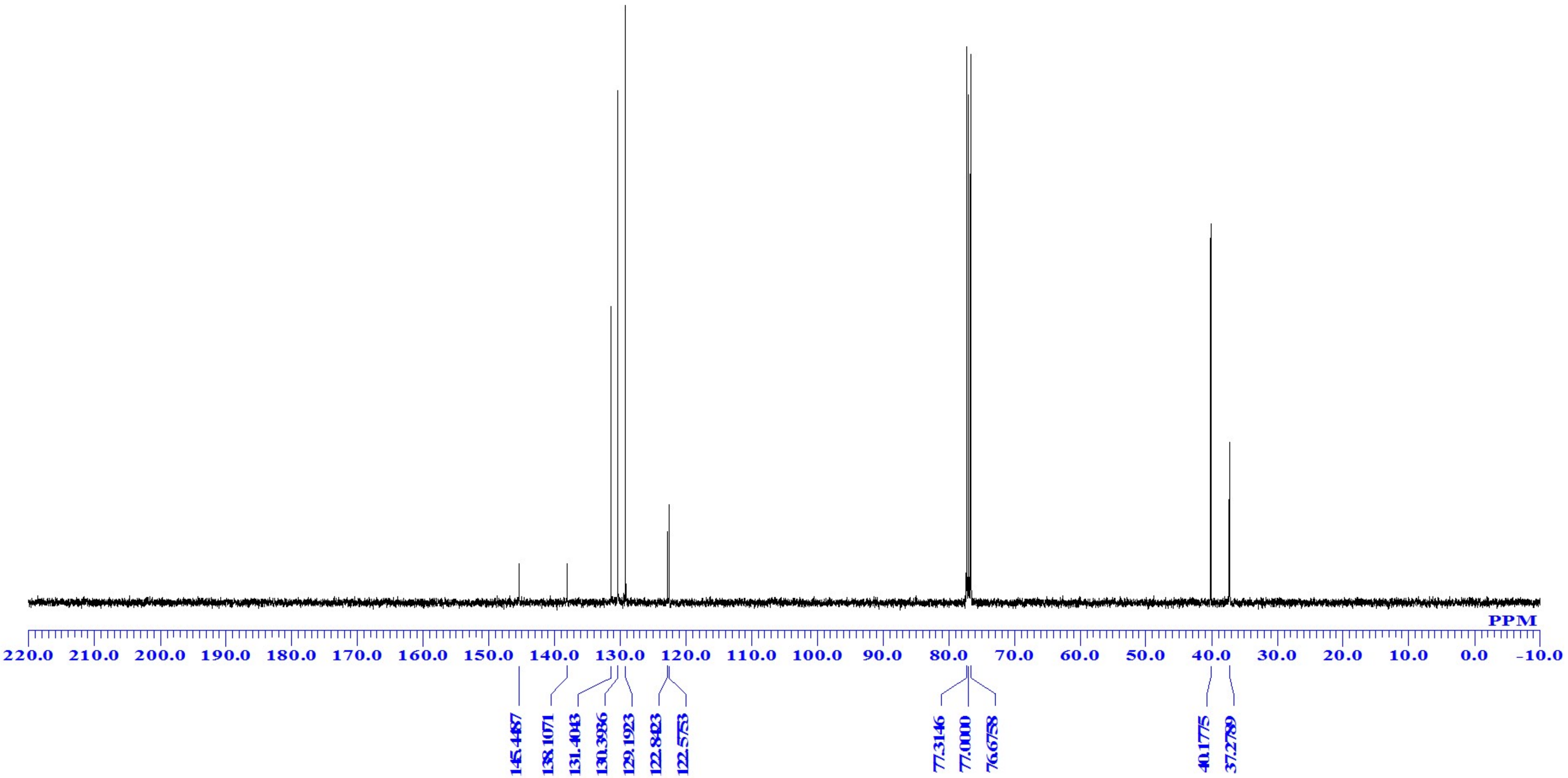

^{1}H NMR spectrum of **1l** (400 MHz, $CDCl_3$)

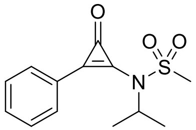

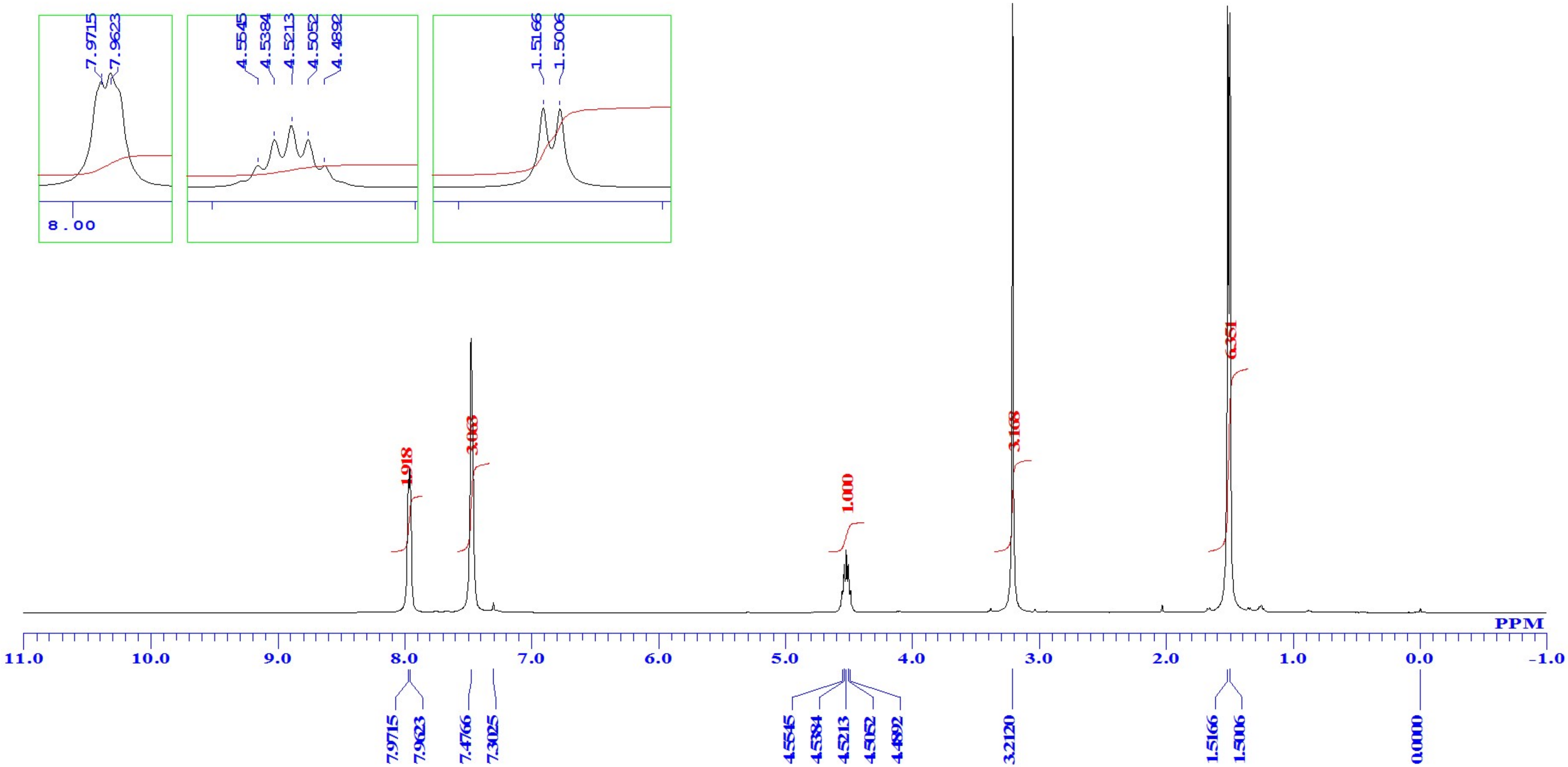

^{13}C NMR spectrum of **1l** (100 MHz, $CDCl_3$)

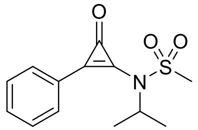

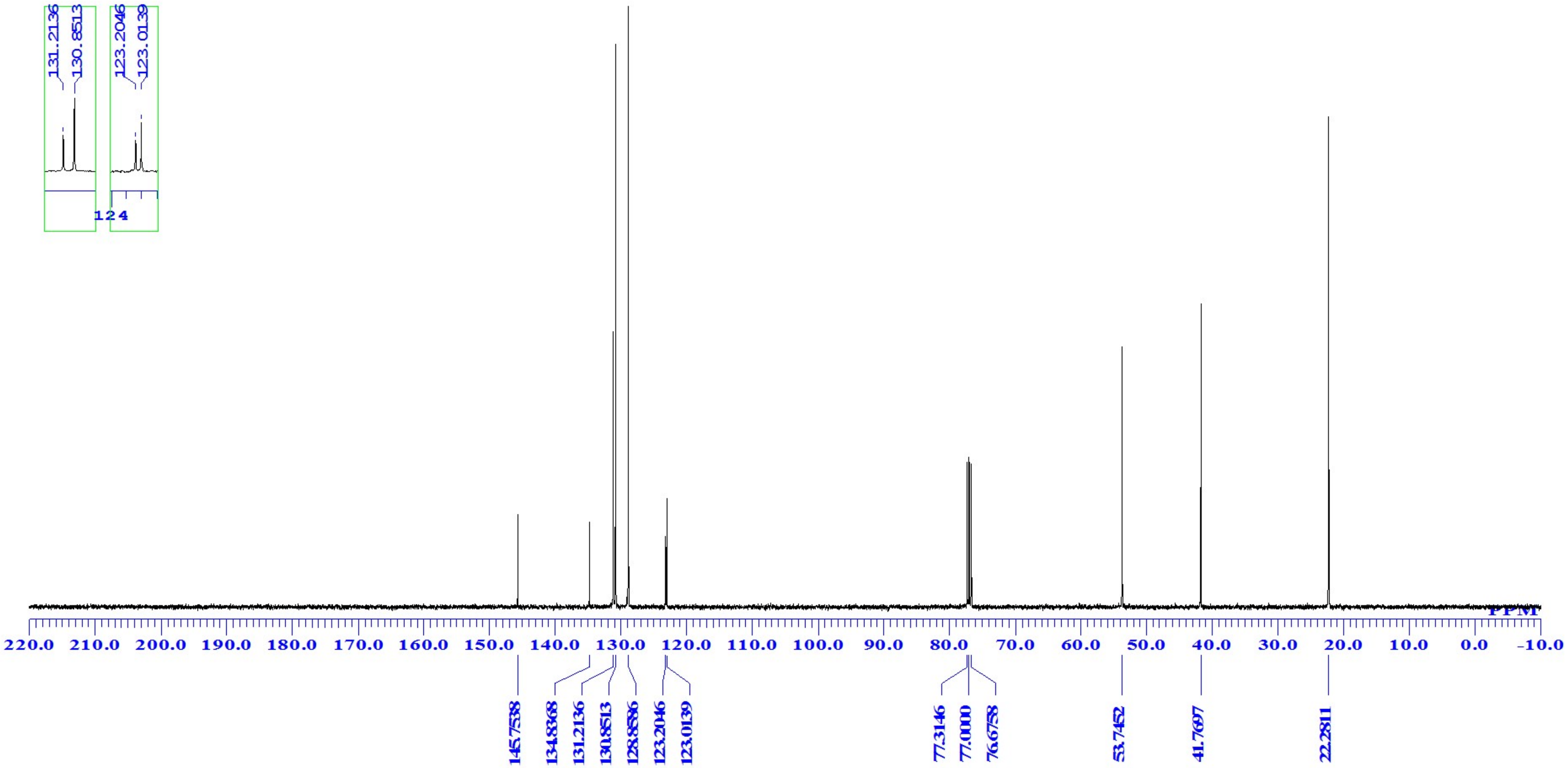

^{1}H NMR spectrum of **1m** (400 MHz, $CDCl_3$)

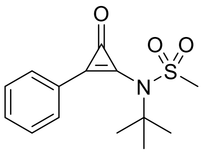

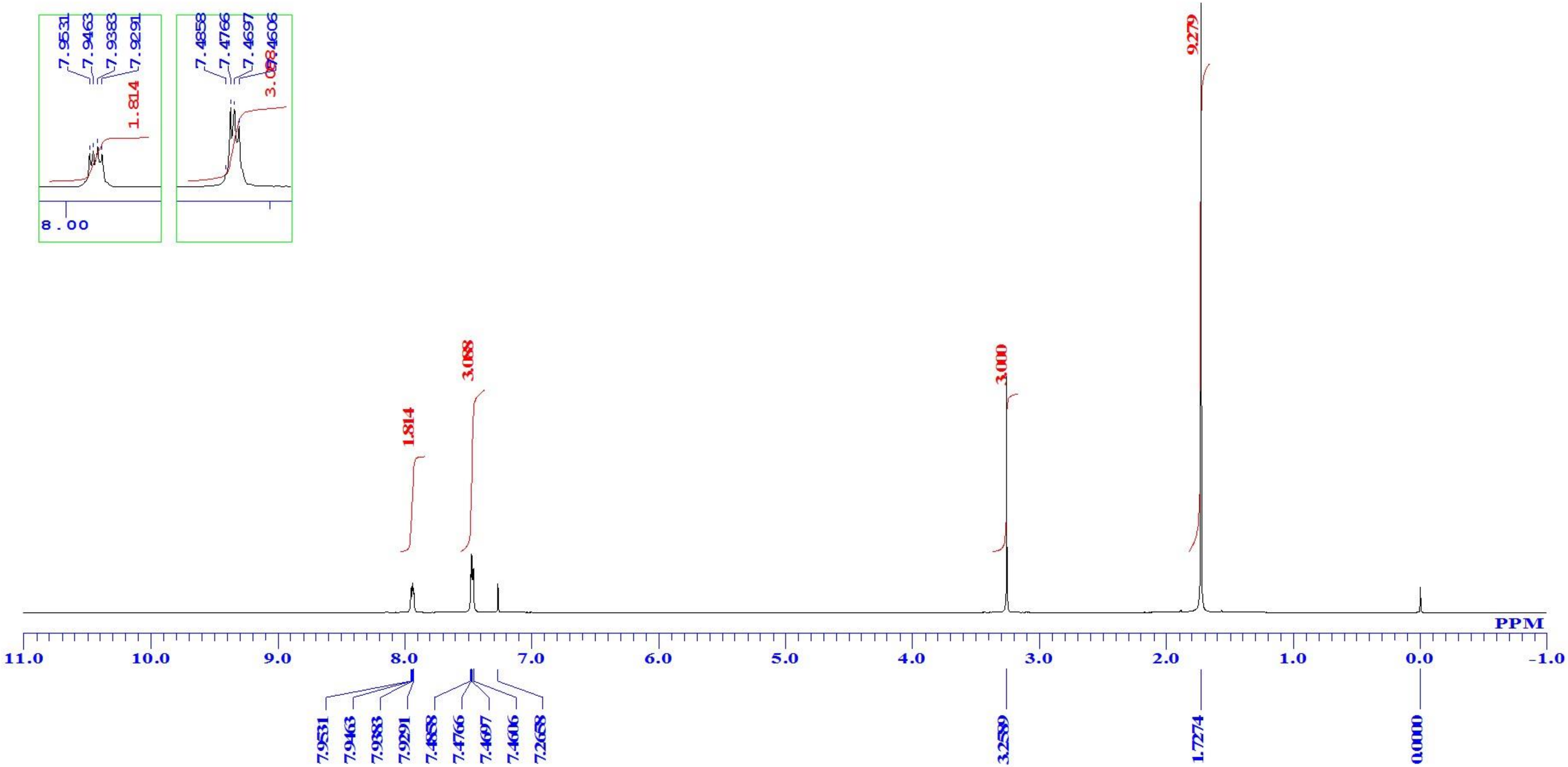

^{13}C NMR spectrum of **1m** (100 MHz, $CDCl_3$)

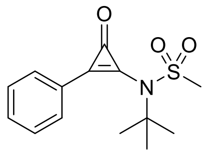

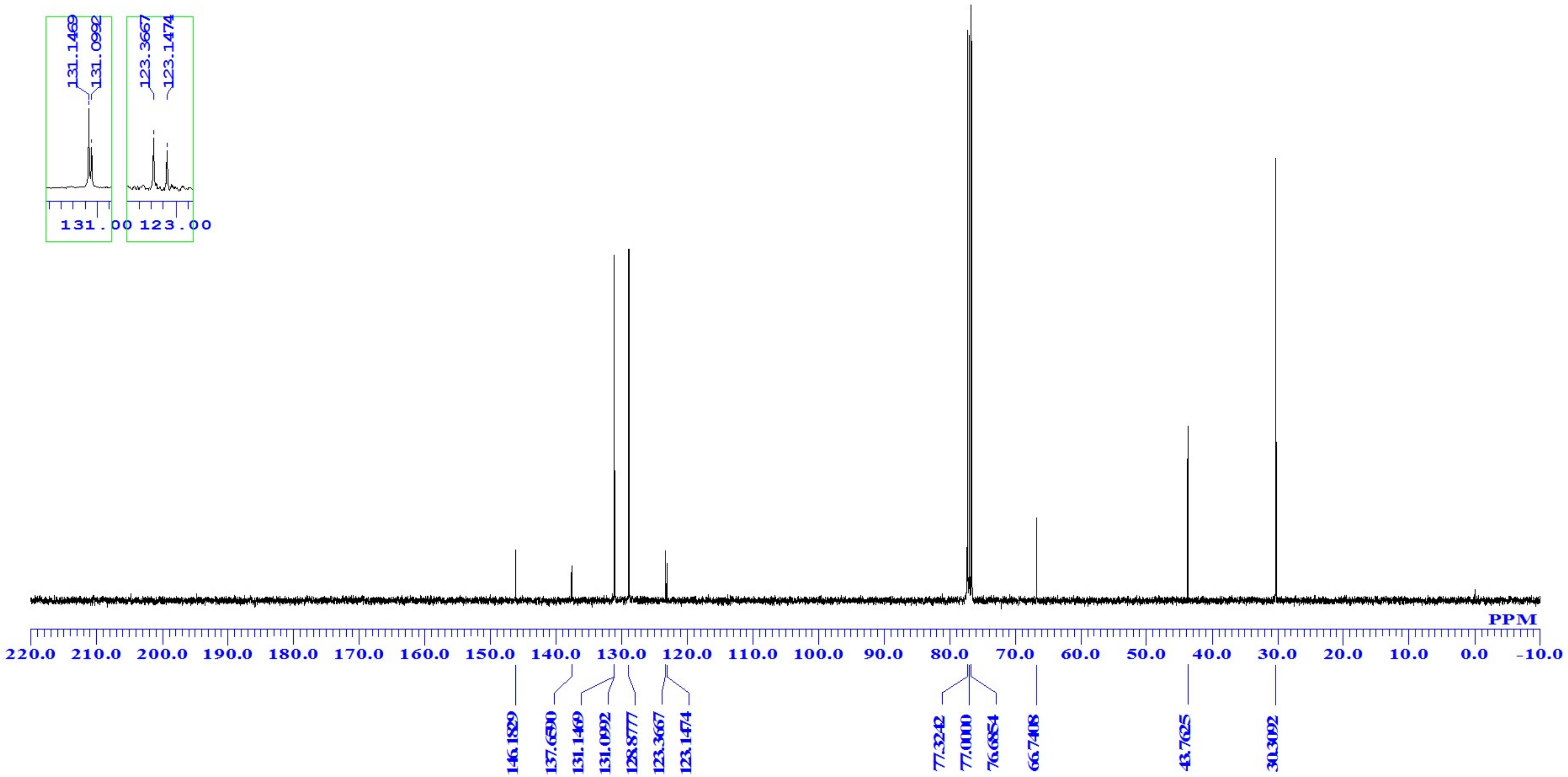

^{1}H NMR spectrum of **1n** (400 MHz, $CDCl_3$)

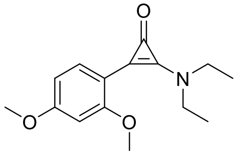

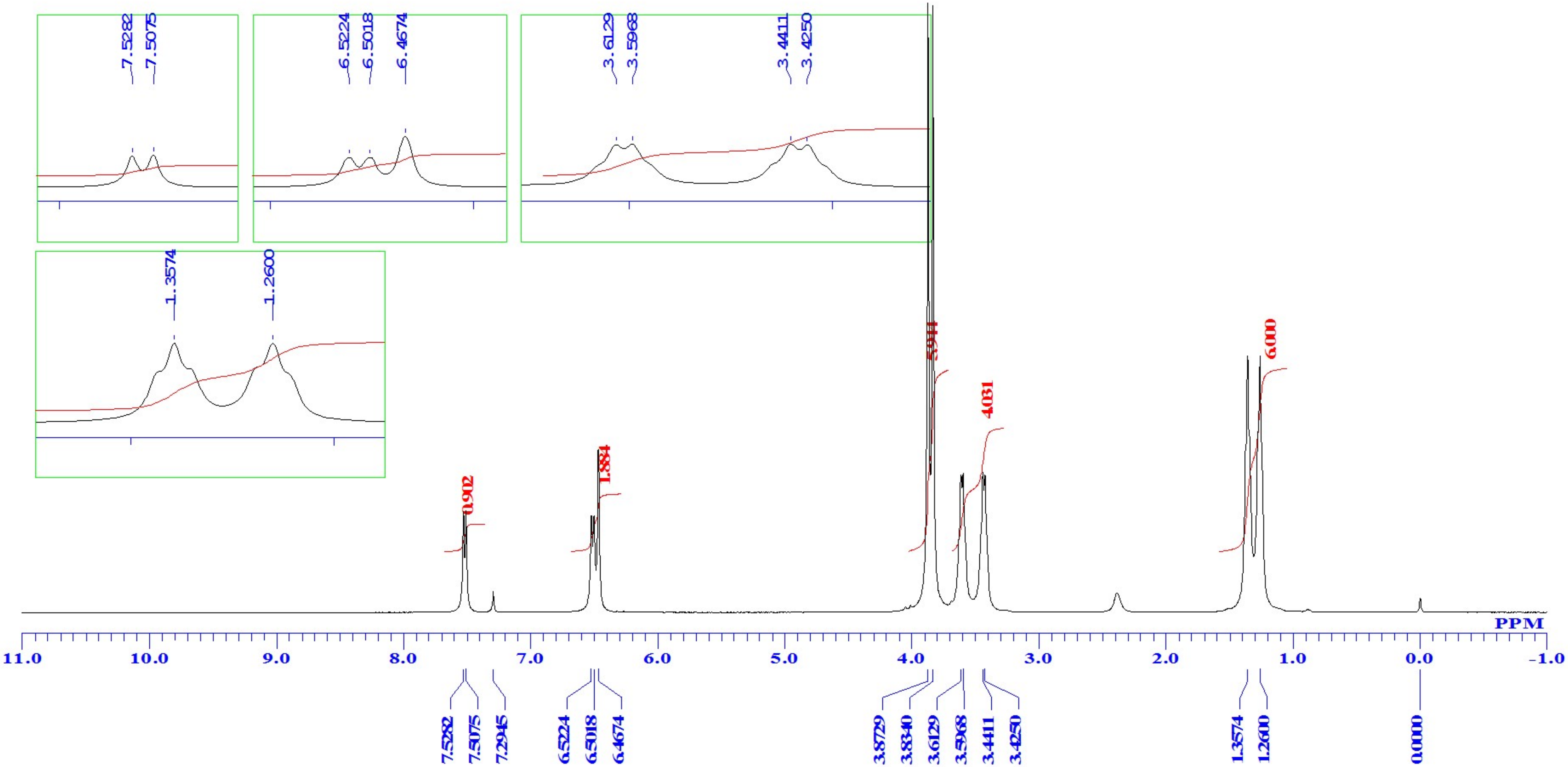

^{13}C NMR spectrum of **1n** (100 MHz, $CDCl_3$)

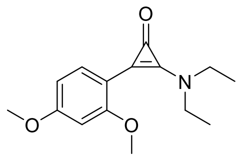

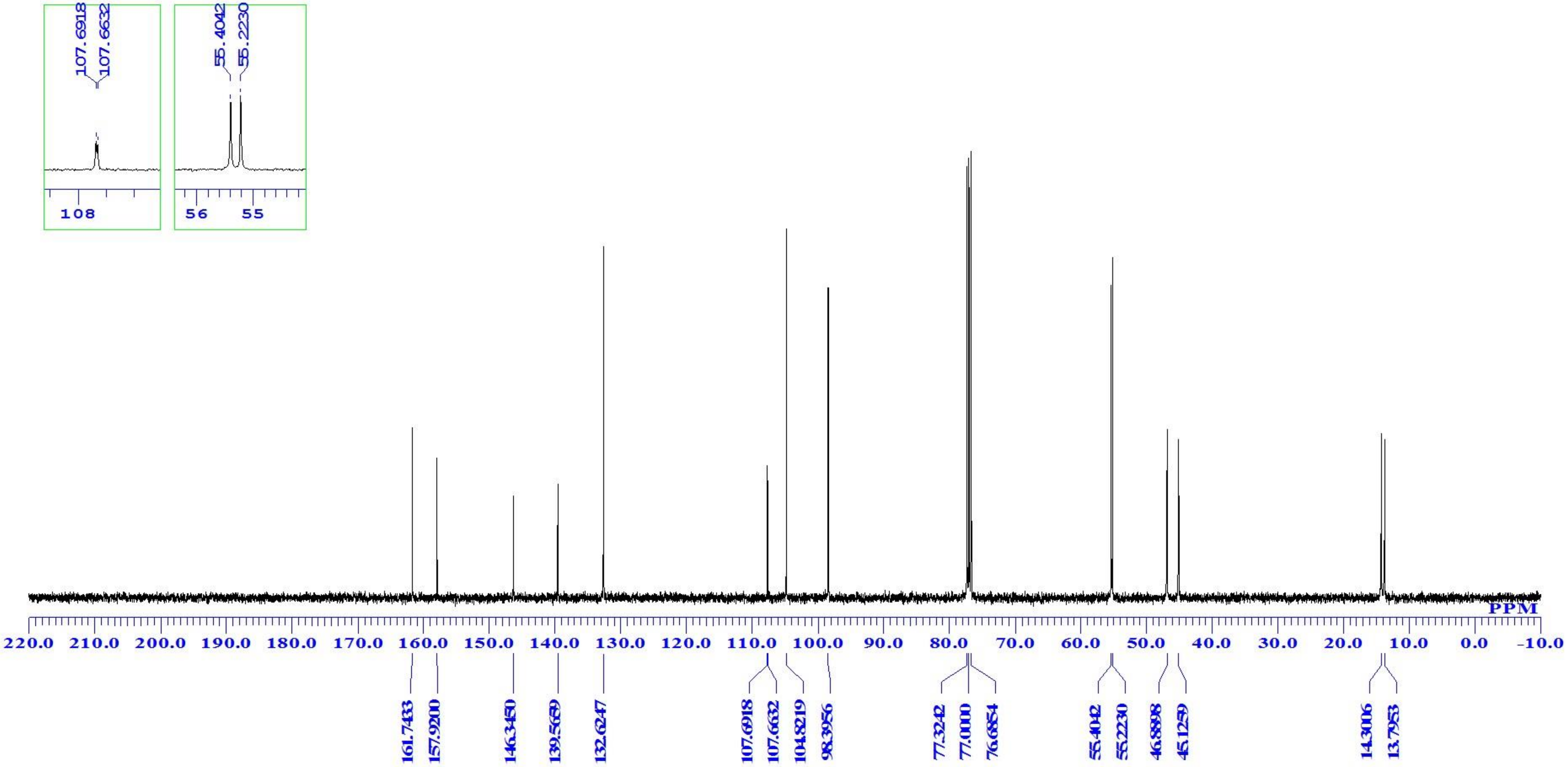

^{1}H NMR spectrum of **S3** (400 MHz, $CDCl_3$)

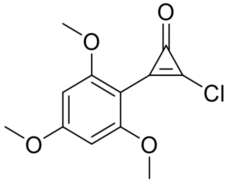

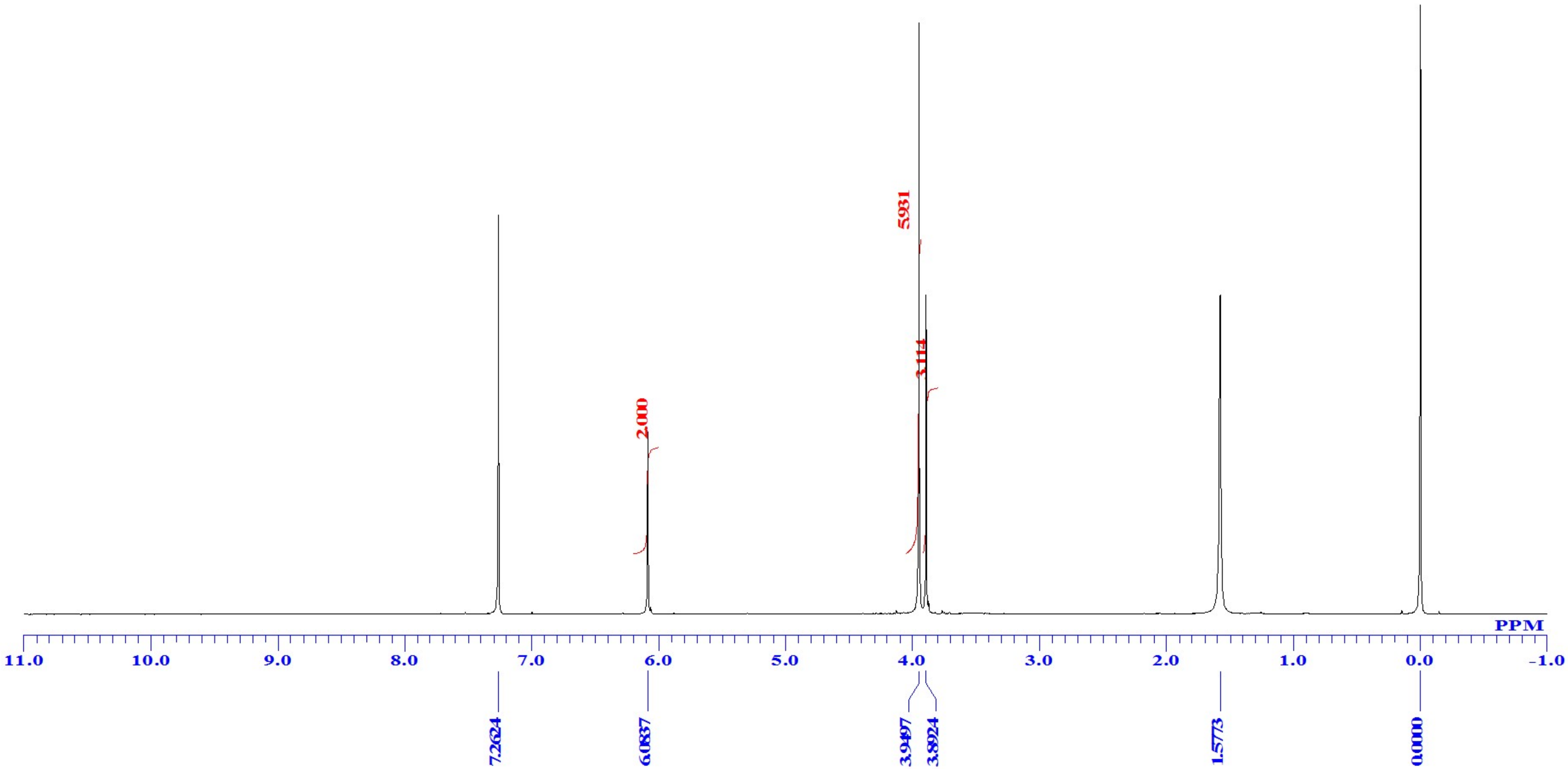

^{13}C NMR spectrum of **S3** (100 MHz, $CDCl_3$)

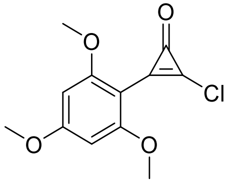

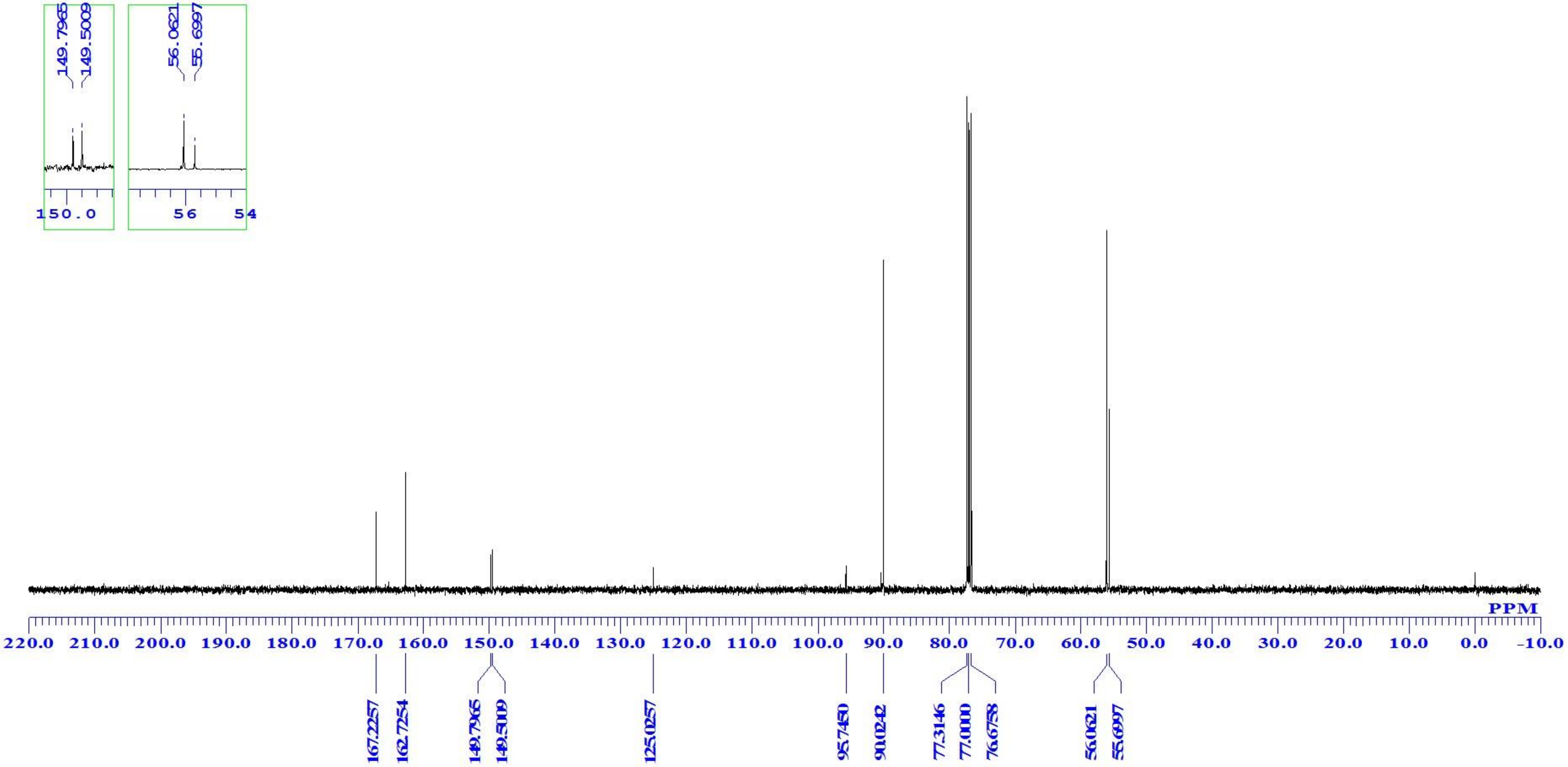

^{1}H NMR spectrum of **1o** (400 MHz, CDCl3)

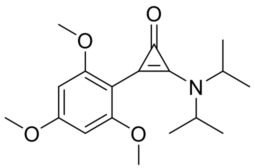

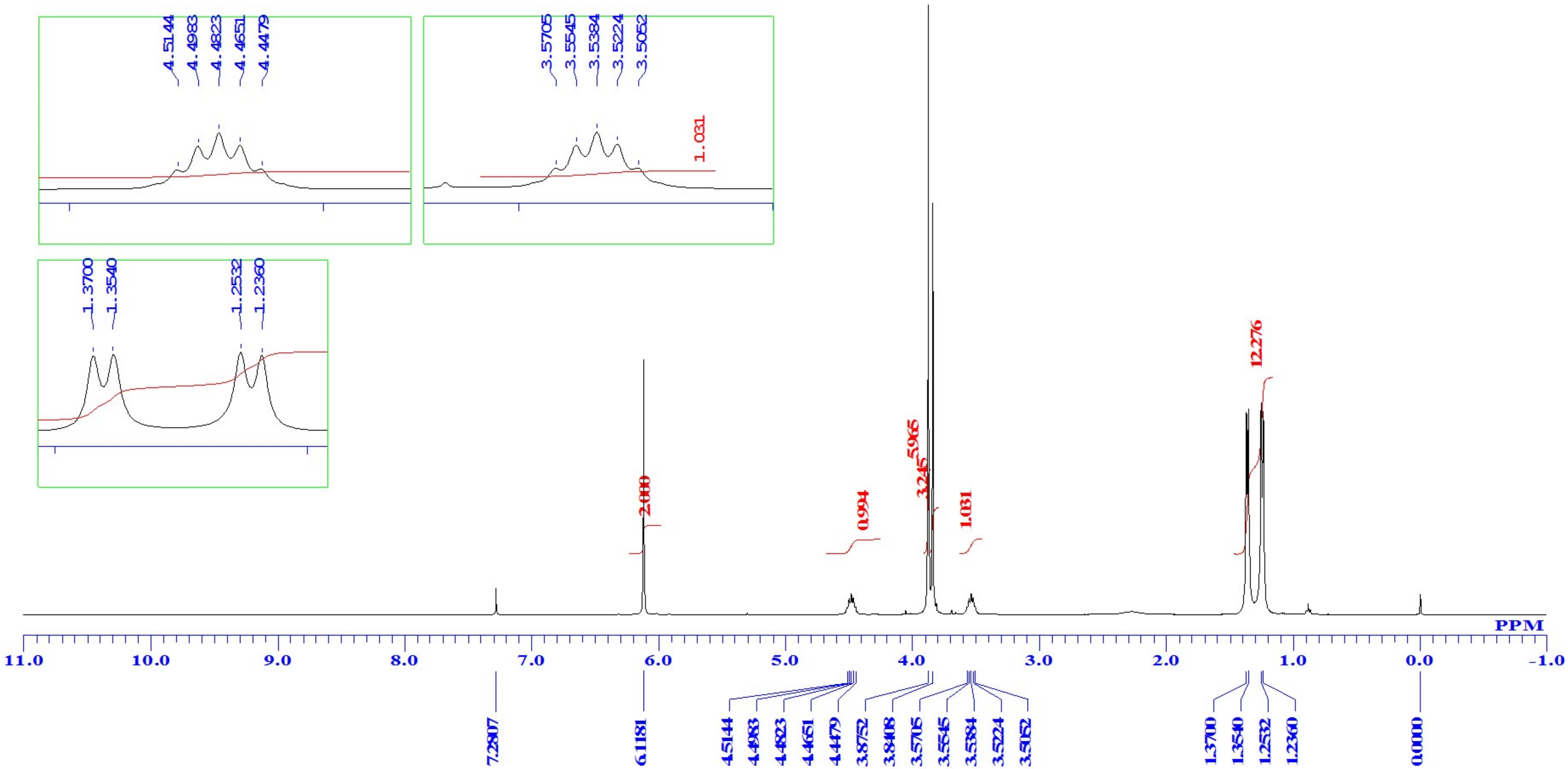

^{13}C NMR spectrum of **1o** (100 MHz, $CDCl_3$)

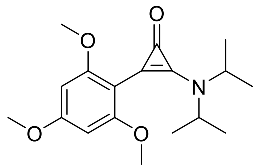

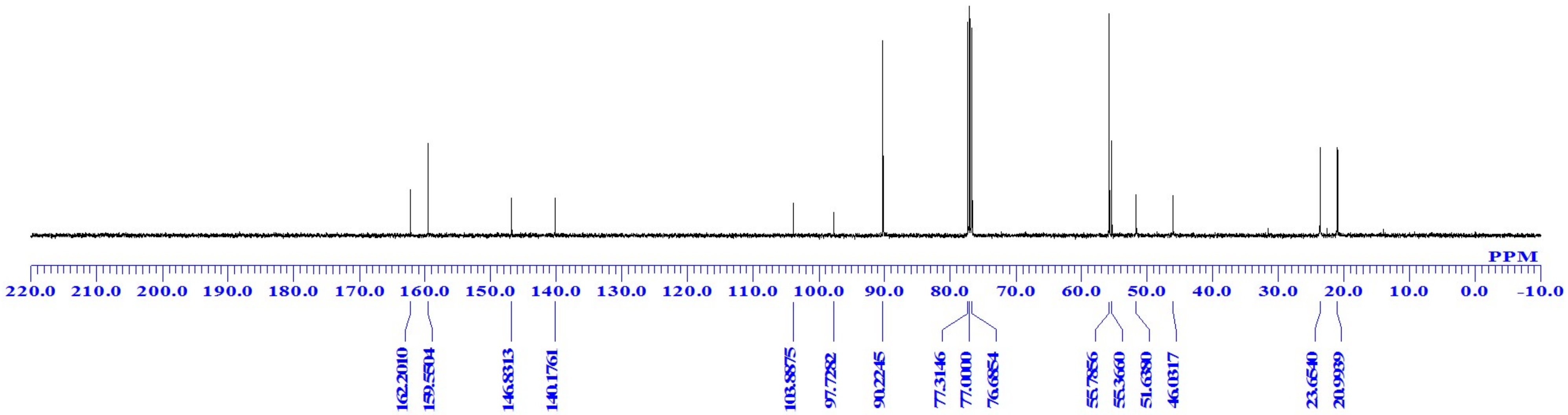

^{1}H NMR spectrum of **1p** (400 MHz, $CDCl_3$)

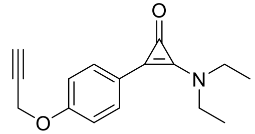

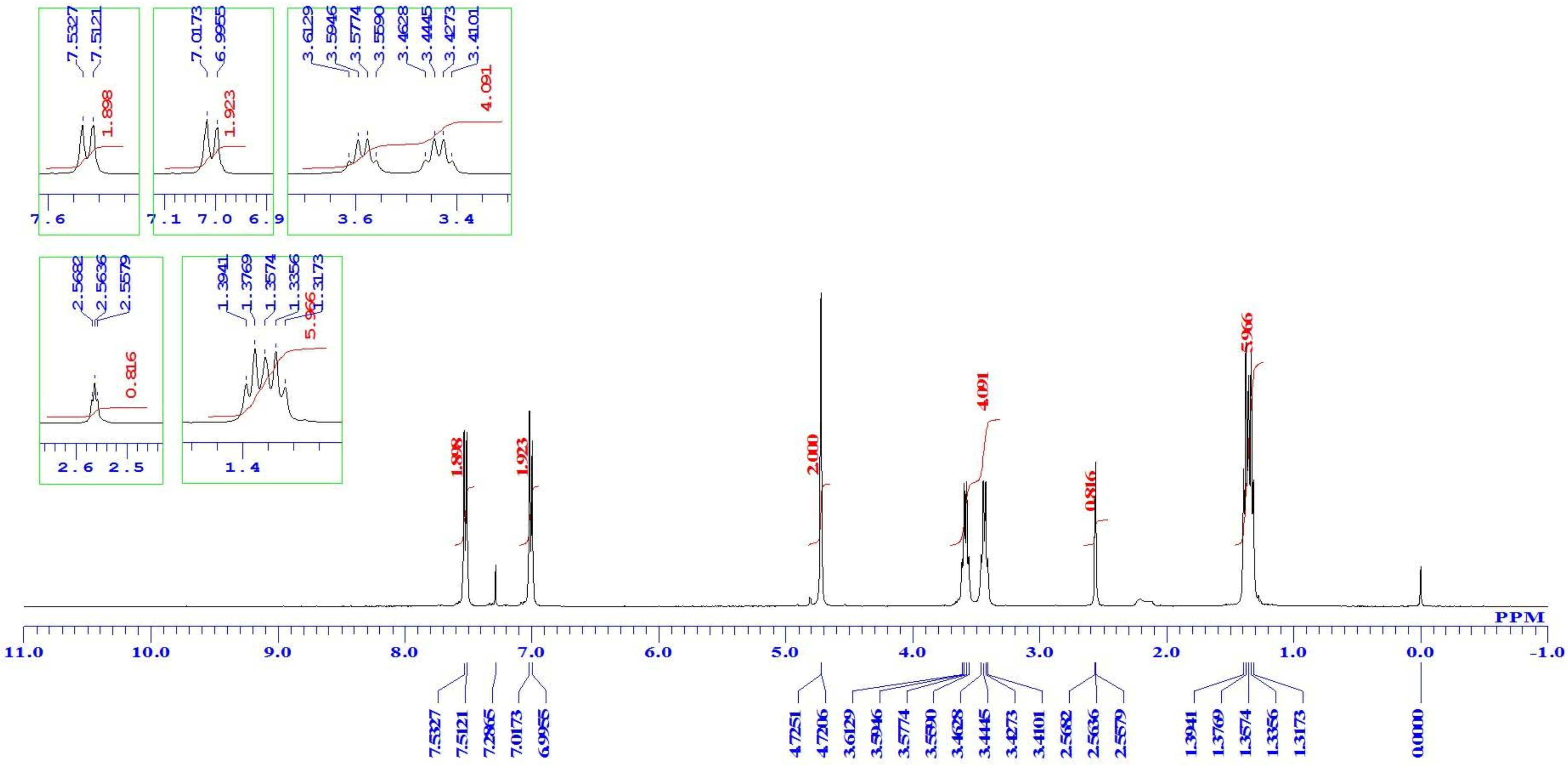

^{13}C NMR spectrum of **1p** (100 MHz, $CDCl_3$)

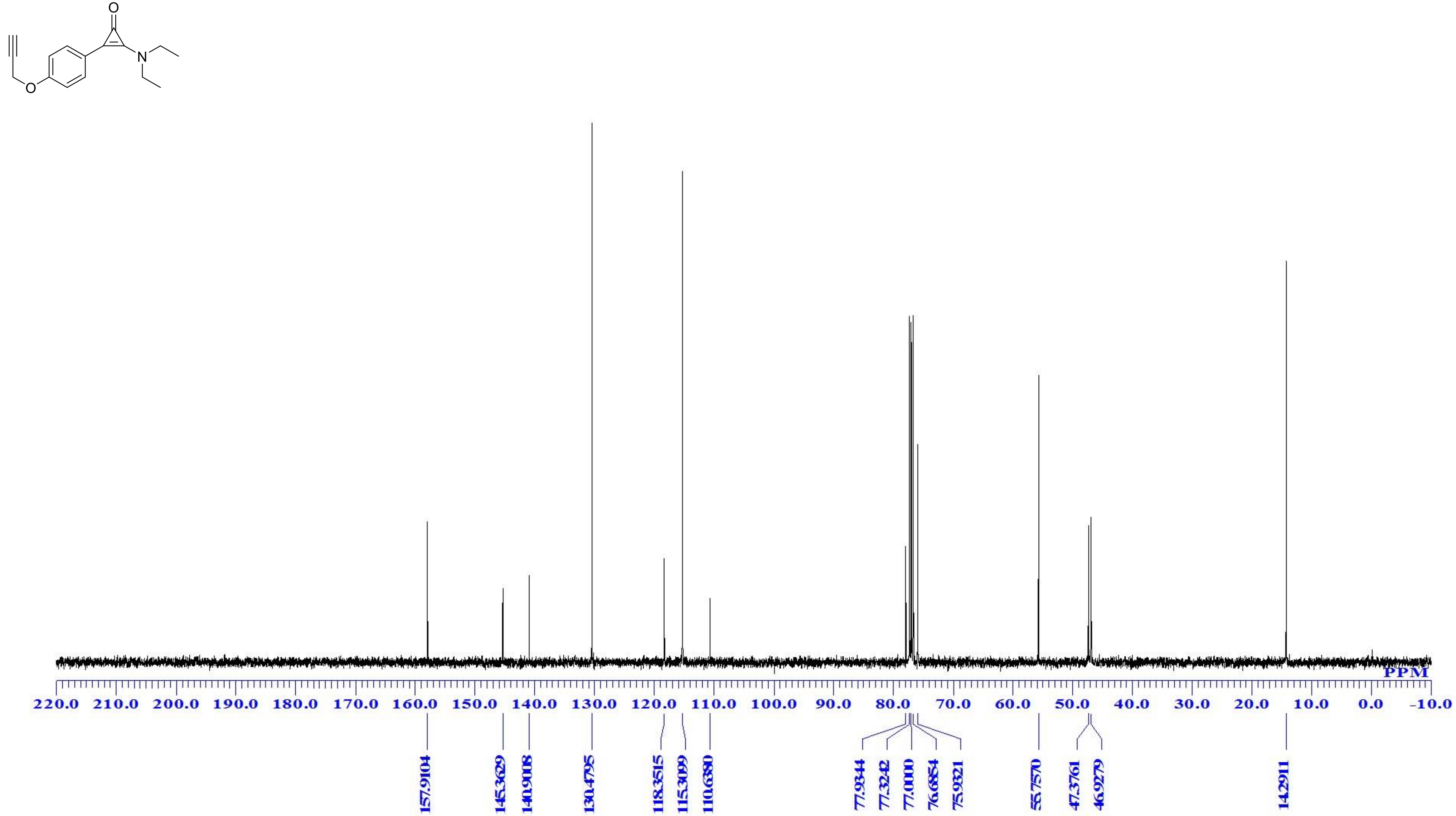

^{1}H NMR spectrum of **1r** (400 MHz, $CDCl_3$)

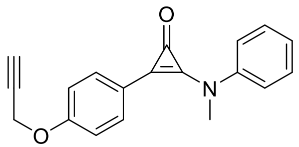

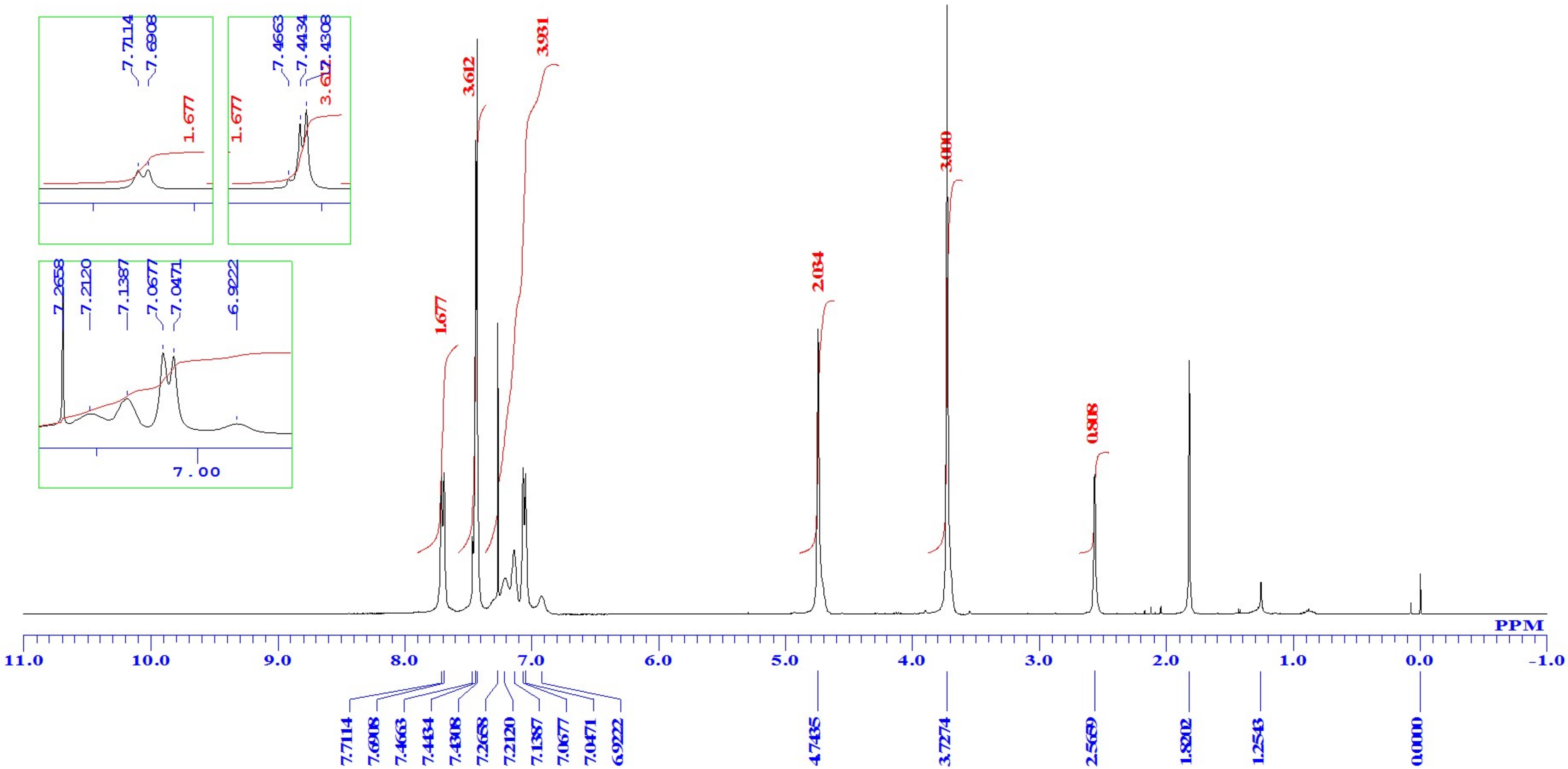

^{13}C NMR spectrum of **1r** (100 MHz, $CDCl_3$)

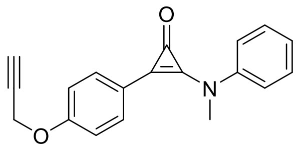

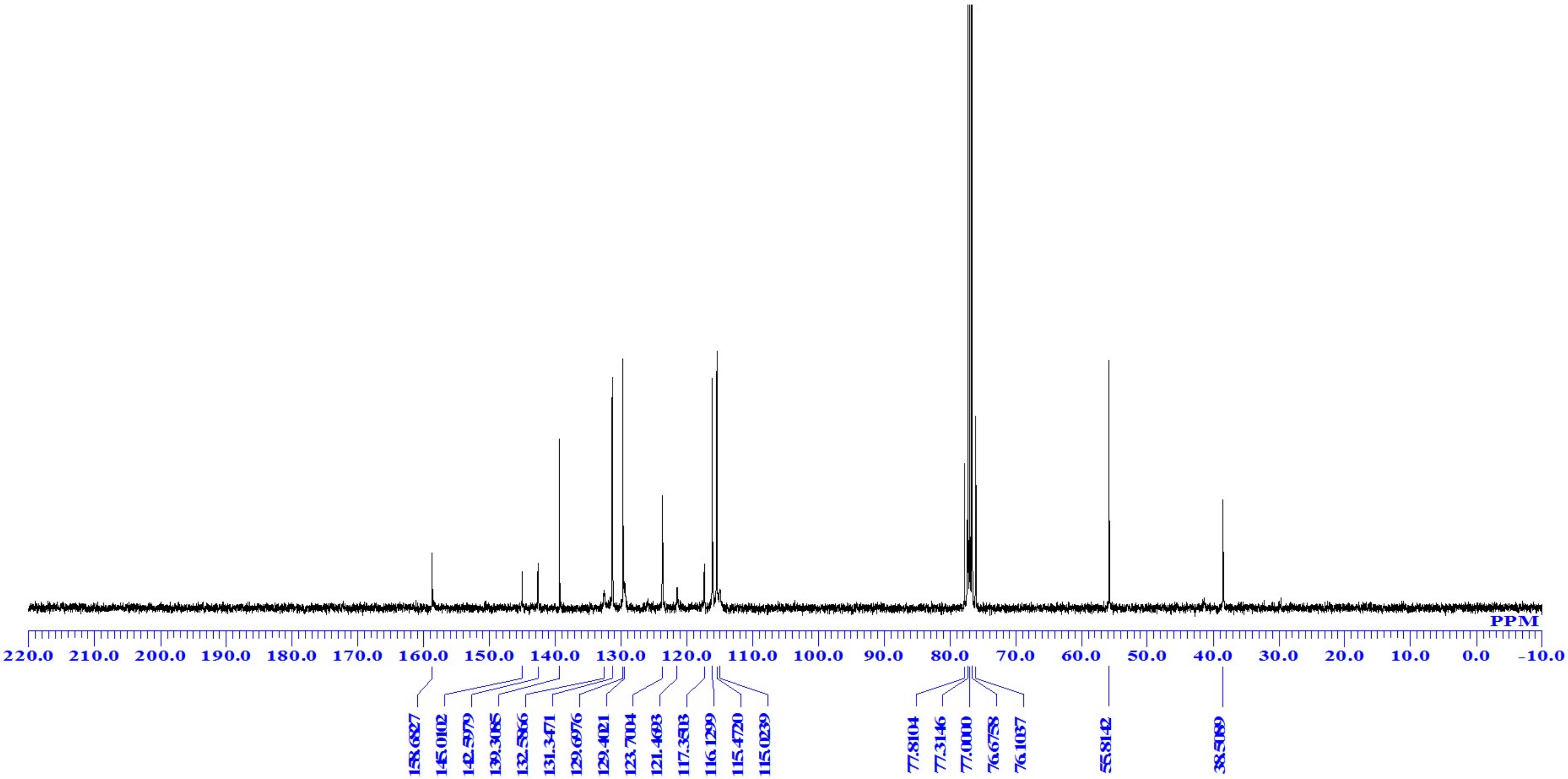

^1H NMR spectrum of **1s** (400 MHz, $CDCl_3$)

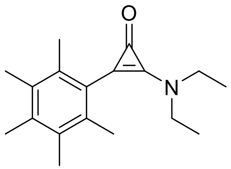

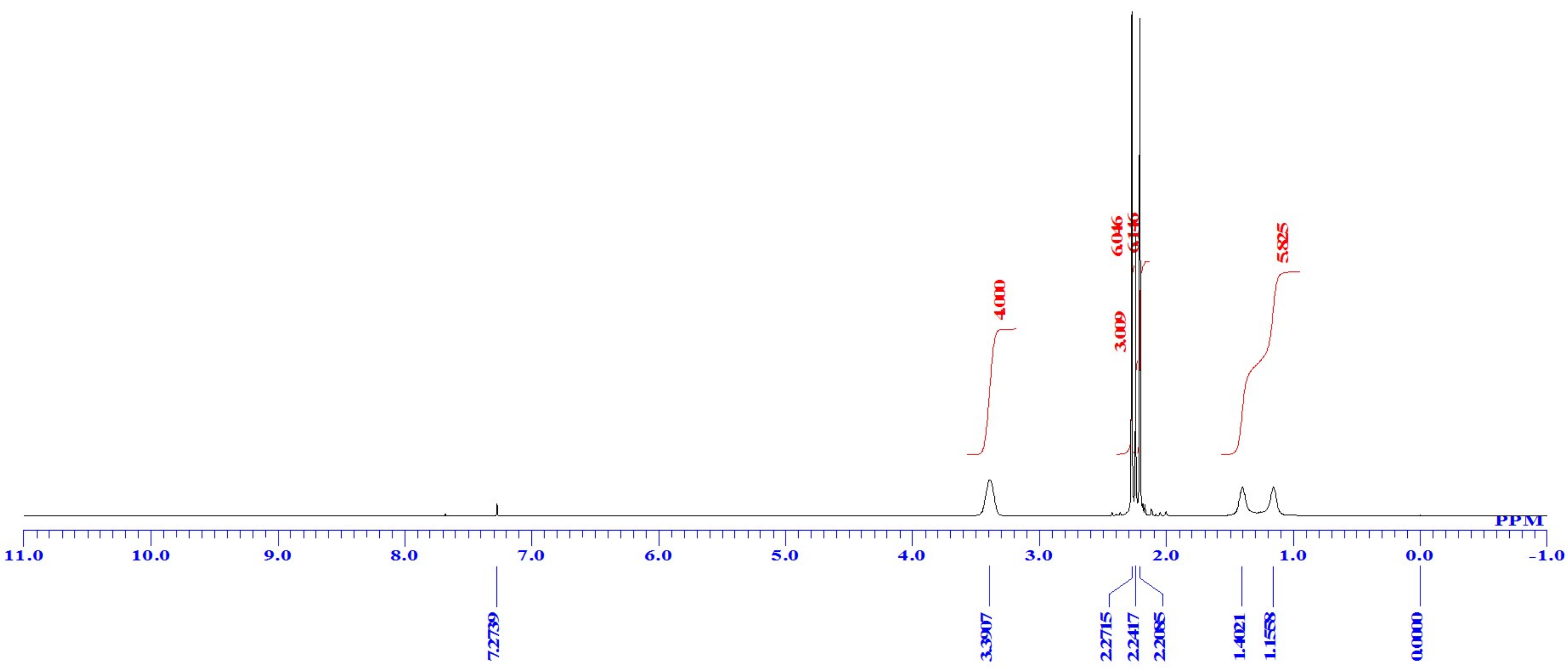

^{13}C NMR spectrum of **1s** (100 MHz, $CDCl_3$)

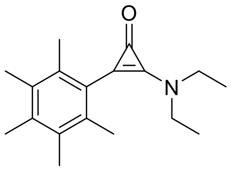

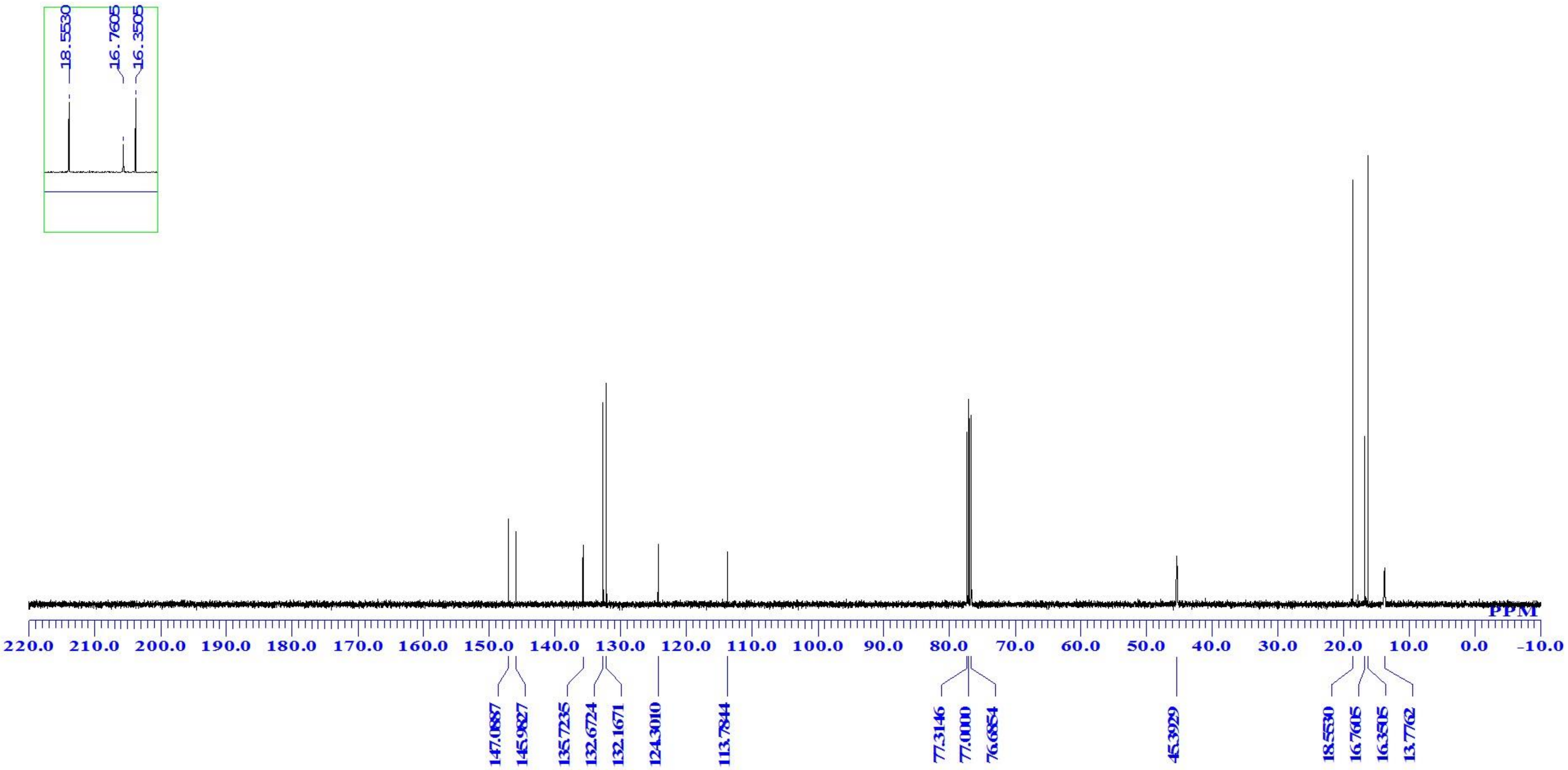

^{1}H NMR spectrum of **1t** (400 MHz, $CDCl_3$)

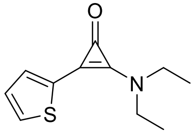

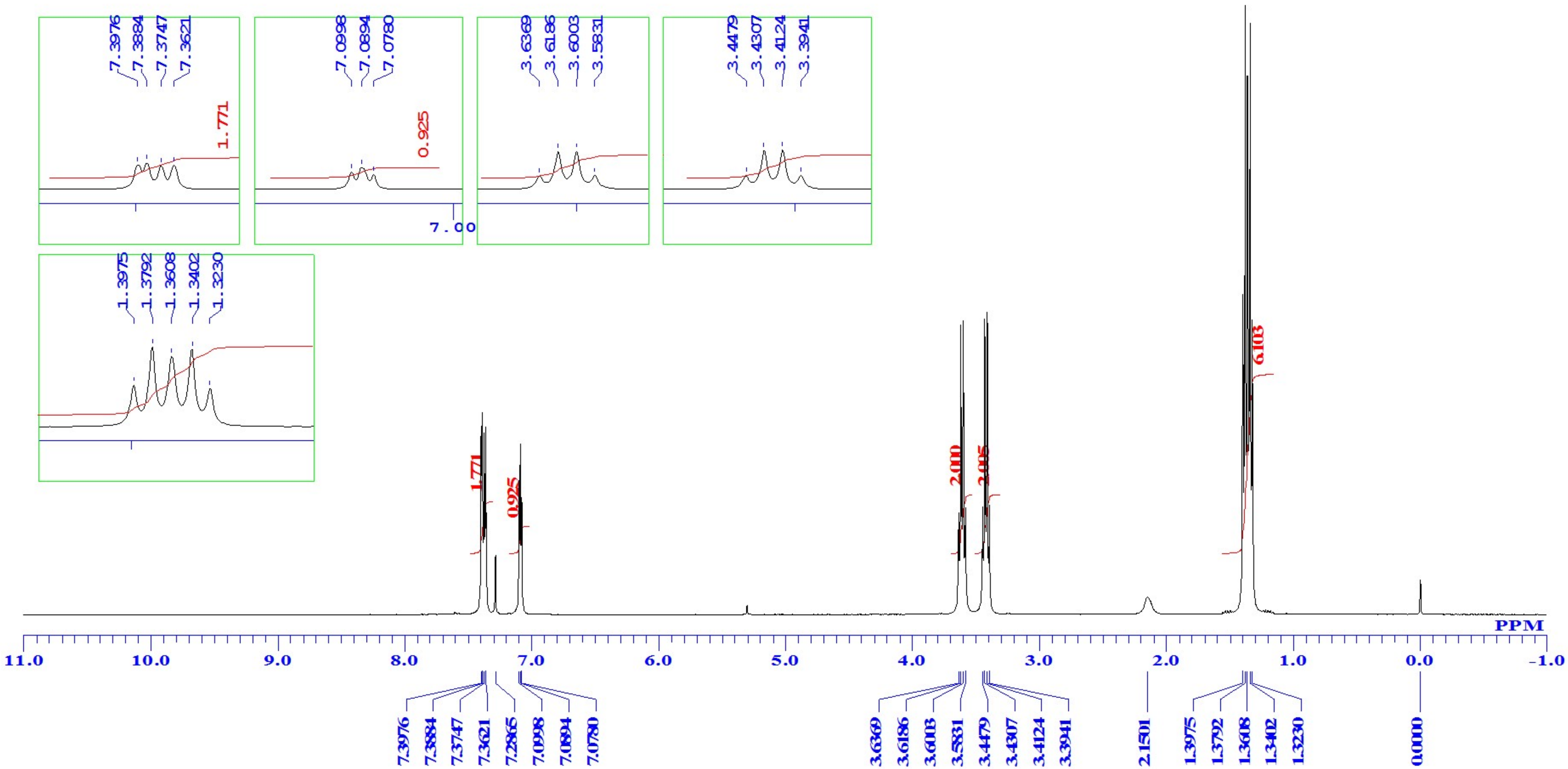

^{13}C NMR spectrum of **1t** (100 MHz, $CDCl_3$)

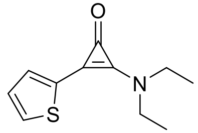

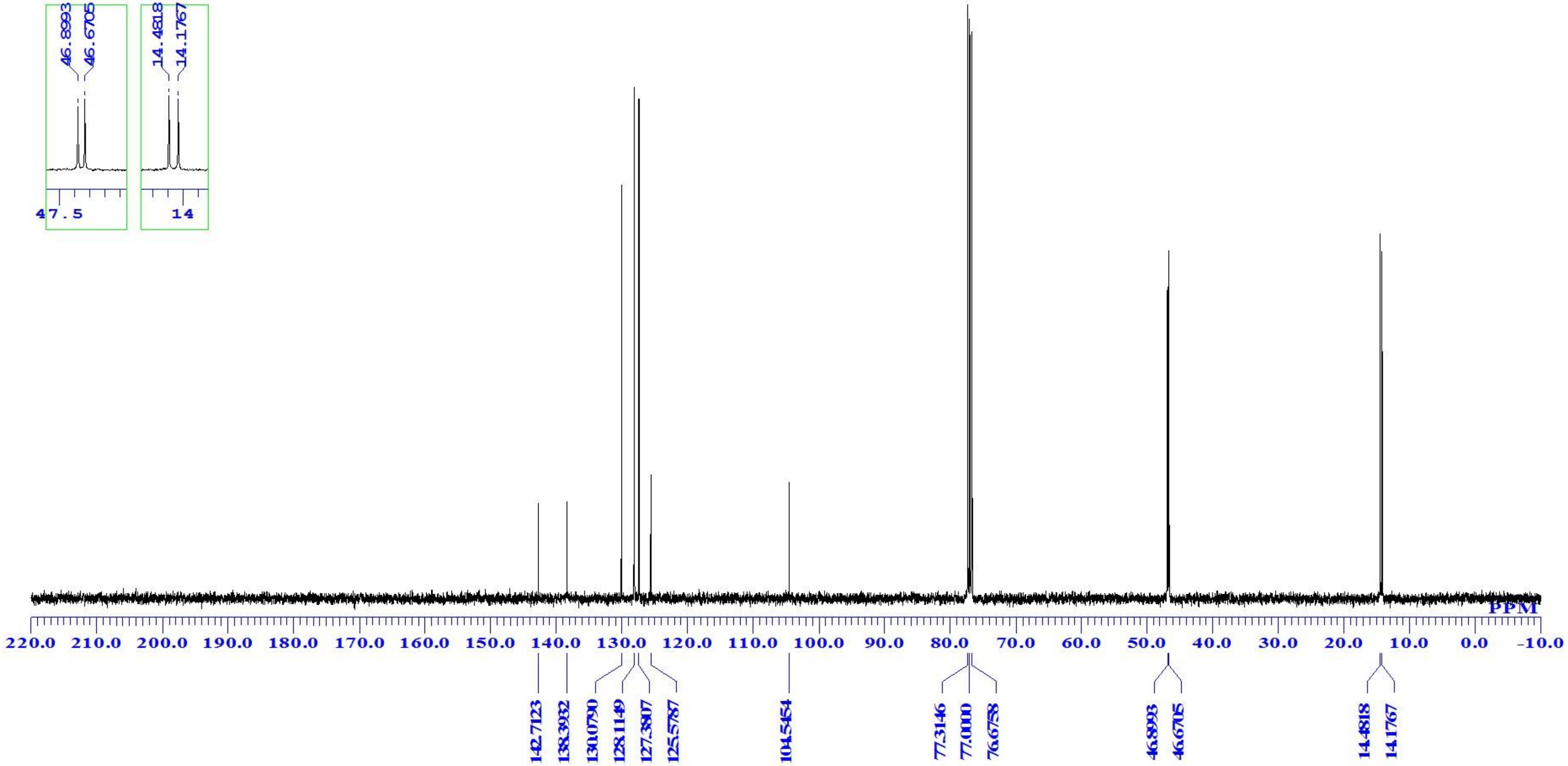

^{1}H NMR spectrum of **2n** (400 MHz, $CDCl_3$)

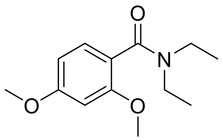

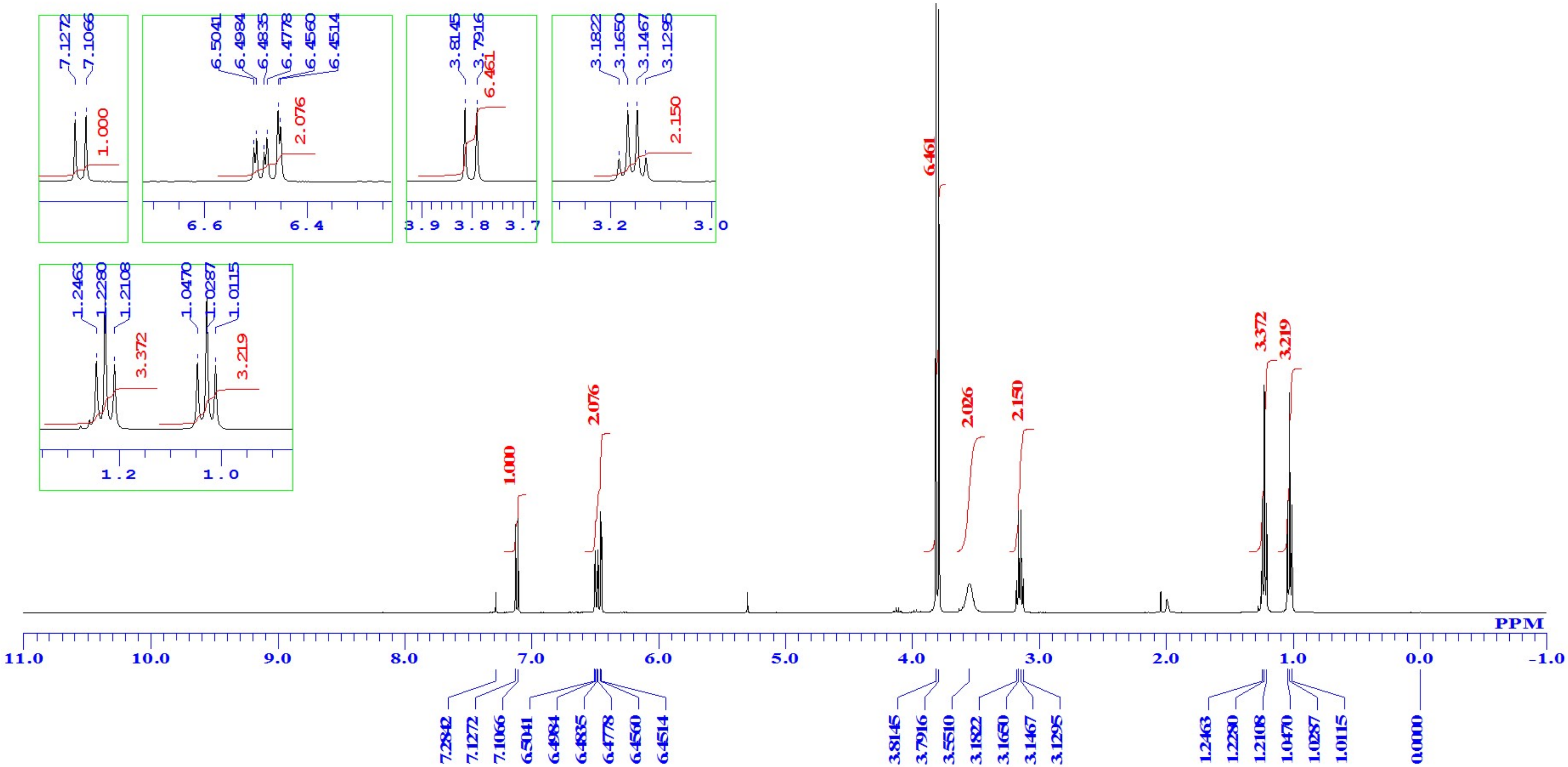

^{13}C NMR spectrum of **2n** (100 MHz, $CDCl_3$)

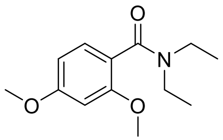

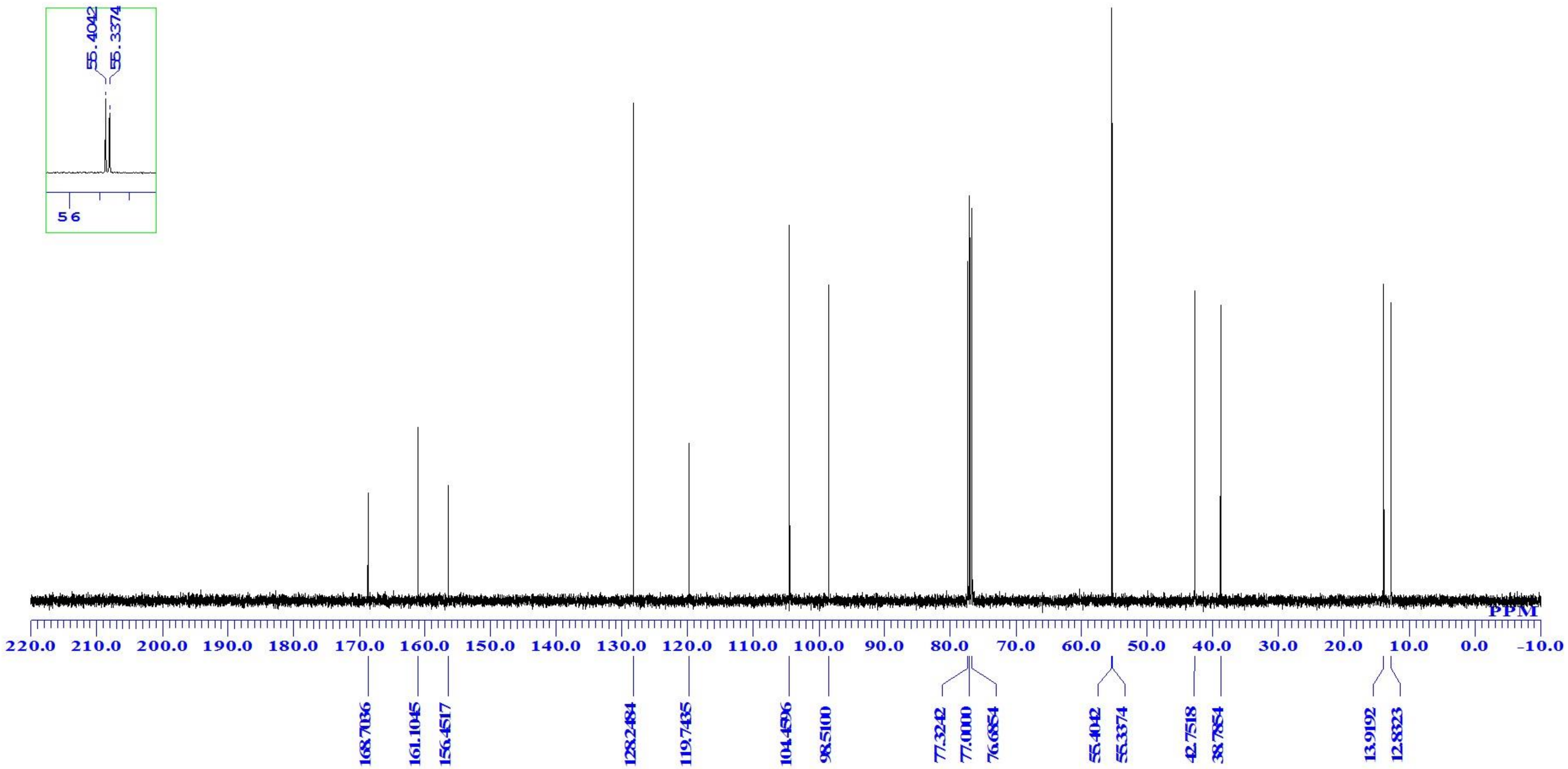

Figure S1. Synthetic procedure and NMR spectra of aminocyclopropenone compounds.
