# Supplementary material for: Discovery of a Novel Aminocyclopropenone Compound That Inhibits BRD4-Driven Nucleoporin NUP210 Expression and Attenuates Colorectal Cancer Growth
Source: Cells. 2022 Jan 18;11(3):317. doi: 10.3390/cells11030317 (PMC8833887; doi:10.3390/cells11030317)
Supplement: Supplementary file 1 [file cells-11-00317-s001.zip › Supplementary Figure S3-S7.pdf]

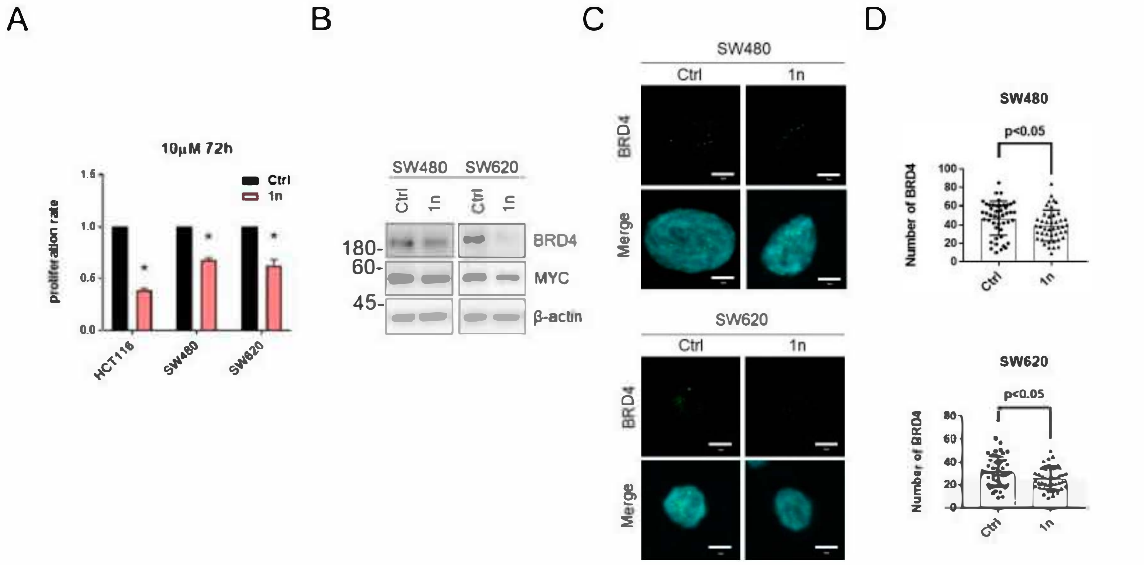

Figure S3. Aminocyclopropenone 1n inhibits SW480 and SW620 colorectal cancer cells growth and in vivo BRD4 assembly respectively. (A) Relative viable number of HCT116, SW480 and SW620 cells after treatment with aminocyclopropenone 1n (72 h, 10 μM). The proliferation rate of control cells is set as 1.0. Data show mean ± SE from three independent experiments (n = 3) and * indicates P <0.05. (B) Western blotting analysis of BRD4 and MYC levels upon aminocyclopropenone 1n administration (24 h, 10 μM) in SW480 and SW620 cells. (C) Immunofluorescence confocal microscopic analysis of BRD4 in SW480 and SW620 cells after aminocyclopropenone 1n treatment (12 h, 10 μM). (D) Number of BRD4 aggregates in the nucleus upon aminocyclopropenone 1n administration (12 h, 10 μM).

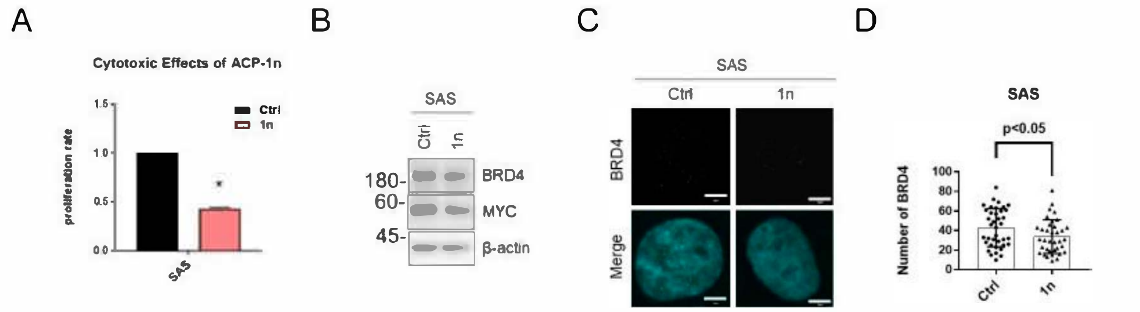

Figure S4. Aminocyclopropenone 1n also inhibits human head and neck squamous cell carcinoma (HNSCC) SAS cells growth and in vivo BRD4 assembly. (A) Relative viable number of SAS cells after treatment with aminocyclopropenone 1n (72 h, 10 μM). The proliferation rate of control SAS cells is set as 1.0. Data show mean ± SE from three independent experiments (n = 3) and * indicates P <0.05. (B) Western blotting analysis of BRD4 and MYC levels upon aminocyclopropenone 1n administration (24 h, 10 μM) in SAS cells. (C) Immunofluorescence confocal microscopic analysis of BRD4 in SAS cells after aminocyclopropenone **1n** treatment (12 h, 10 μM). (D) Number of BRD4 aggregates in the nucleus upon aminocyclopropenone 1n administration (12 h, 10 μM).

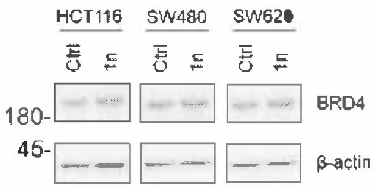

Figure S5. No visible changes of BRD4 protein expression after ACP-1n treatment (12 h, 10 μM) in several CRC cell lines.

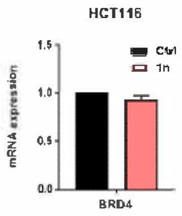

Figure S6. qRT-PCR analysis of BRD4 mRNA in HCT116 cells after aminocyclopropenone 1n treatment (24 h, 10 μM). Expression level of mRNA from control HCT116 cells is set as 1.0. Data show mean ± SE from three independent experiments (n = 3).

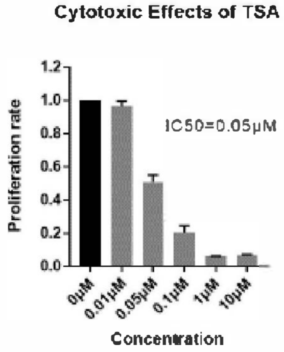

Figure S7. Effect of trichostatin A on viability of HCT116 cells. Data show mean ± SE from three independent ex-pediments (n = 3).
