# Supplementary material for: Discovery of a Novel Aminocyclopropenone Compound That Inhibits BRD4-Driven Nucleoporin NUP210 Expression and Attenuates Colorectal Cancer Growth
Source: Cells. 2022 Jan 18;11(3):317. doi: 10.3390/cells11030317 (PMC8833887; doi:10.3390/cells11030317)
Supplement: Supplementary file 1 [file cells-11-00317-s001.zip › Supplementary table.pdf]

Supporting Information

Discovery of a Novel Aminocyclopropenone Compound that Inhibits BRD4-driven Nucleoporin NUP210 Expression and Attenuates Colorectal Cancer Growth

Hiroya Kondo [1], Kenji Mishiro [2], Yuki Iwashima [3], Qiu Yujia [4], Akiko Kobayashi [2], Keesiang Lim [4], Toshinari Minamoto [5], Kazuma Ogawa [2], Munetaka Kunishima [6], Masaharu Hazawa [2,3,4,*] and Richard W. Wong [2,3,4,*]

1 Division of Transdisciplinary Sciences, Graduate School of Frontier Science Initiative, Kanazawa University, Kakuma-machi, Kanazawa, Ishikawa 920-1192, Japan; hiro4015@stu.kanazawa-u.ac.jp

2 Institute for Frontier Science Initiative, Kanazawa University, Kakuma-machi, Kanazawa, Ishikawa 920-1192, Japan; mishiro@p.kanazawa-u.ac.jp (K.M.); akoba@staff.kanazawa-u.ac.jp (A.K.); kogawa@p.kanazawa-u.ac.jp (K.O.)

3 Laboratory of Molecular Cell Biology, School of Natural System, Institute of Science and Engineering, Kanazawa University, Kakuma-machi, Kanazawa, Ishikawa 920-1192, Japan; yuki090606@stu.kanazawa-u.ac.jp

4 WPI-Nano Life Science Institute, Kanazawa University, Kakuma-machi, Kanazawa, Ishikawa 920-1192, Japan; porthos0616@gmail.com (Q.Y.); limkeesiang@staff.kanazawa-u.ac.jp (K.L.)

5 Division of Translational and Clinical Oncology, Cancer Research Institute, Kanazawa University, Kanazawa, Ishikawa 920-0934, Japan; minamoto@staff.kanazawa-u.ac.jp

6 Faculty of Pharmaceutical Sciences, Institute of Medical, Pharmaceutical, and Health Sciences, Kanazawa University, Kakuma-machi, Kanazawa 920-1192 Japan; kunisima@p.kanazawa-u.ac.jp

* Correspondence: masaharu.akj@gmail.com (M.H.); rwong@staff.kanazawa-u.ac.jp (R.W.); Tel.: +81-076-264-6253

Table of Contents

Antibodies (Table S1) S2

Primers sequences for qRT-PCR (Table S2).......... S3-S4

Table S1. Antibodies

Antibody	Company	Catalog #
β-actin	Cell Signaling Technology	4967S
BRD4	Abcam	ab128874
BRD4	Cell Signaling Technology	13440s
NUP58	Sigma	HPA039360
mrnp41 (H-3)	Santa Cruz Biotechnology	sc-393252
NUP37	Santa Cruz Biotechnology	sc-109348
MYC	Wako	017-21871
NUP210	Santa Cruz Biotechnology	sc-79500
Goat anti-Rabbit IgG (H+L), Alexa Fluor 488	Thermo Fisher Scientific	A11034
anti-Mouse IgG	Cell Signaling Technology	7076S
anti-Rabbit IgG	Cell Signaling Technology	7074S
anti-Goat IgG	Invitrogen	#PA1-28664

Table S2. Primer sequences used for qRT-PCR.

Name	Sequence
NUP35-F	CTAGCCCAGGACTTGGATCA
NUP35-R	ACTCTAGCCTGGGCAACTGA
NUP93-F	GTTTGAAGAGGCAGCAAAGC
NUP93-R	ACAGGGCTCAGCAGTTTGTT
NUP155-F	TGAGAATCCCAGCCAAGTTT
NUP155-R	TGGAGCTCAACAAGGTAACCA
NUP188-F	ATAGGCACCTGGTGGATGAG
NUP188-R	CAGGATGAGGGCACTGAAGTA
NUP205-F	TAGACGGGGAAAGACATGGA
NUP205-R	TGCTCCATCAGCTCATCTGT
NUP54-F	TGCAGAATCATTTTGGAGCA
NUP54-R	ATGGCTAAGGCCTTCCTGTT
NUP58-F	TGGGAGGTTCACTTTTCCAG
NUP58-R	TTGCCTGCAGTTGAAGTAGC
NUP62-F	ACATCGATGCACAGCTCAAG
NUP62-R	ACTGCAGTGAGTCCATGTGC
NUP98-F	CAAAGAATACCGGCCTGAAA
NUP98-R	GGATGCTCCTCCTCCTCTTC
RAE1-F	TTGCAGGATCATGGGCTAAT
RAE1-R	GGCTTTTGGAATGGTCTGTC
NUP37-F	TCAGATCTTCAGGATAAAAATGAA
NUP37-R	GGGATCAAACACCAAACCAT
NUP43-F	GAATTGTCGCTGCTTCATCA
NUP43-R	TCCACTGCTGGTTGACTGAC
NUP85-F	AGGGGAGATGCTGGTATGTG
NUP85-R	CAACTTGGCACCATCTCTGA
NUP96-F	ACTGGGACAGCATTTGGAAC
NUP96-R	ATGAAGGTGCACTGGTTGTG
NUP107-F	TCAGAGGGATTCACTTGTTCG
NUP107-R	TCCAATTTCATCTTTGGCAAT
NUP133-F	TGCCAAATGTCAGATGCAGT
NUP133-R	GAAAAGTTTGGGACCGTCAA

NUP160-F	CACCAGGACAATTCACAAATG
NUP160-R	CAGTTCCTCGGCAGAAAATC
SEH1-F	GGTGATTGGCATTGTACTGCT
SEH1-R	CACACGCCATACAGATCCAC
SEC13-F	GGCTTGTGCTGGAAAAGATG
SEC13-R	TGGGAGGTATCCACAGTGTT
ELYS-F	TGTGCTTCGTGGAAAGTTTG
ELYS-R	CCAAGCAAGCAAGTCCATTT
NUP358-F	CTGACGTGGAGCGGTACAT
NUP358-R	CAGCTTTGCAAAATAGAATCCTT
NUP214-F	CCAAGTGGAAATCACCATCA
NUP214-R	GAGAACTGGAGCAGGAGGAA
NUP88-F	CCTAAAAGATGGGGGAAGAA
NUP88-R	AAAATCTCTCCGCAACTGGA
POM121-F	CCTTGATGGCCAGGAAAATA
POM121-R	TGTCCACTGCCACTGCTATC
NDC1-F	AGGTCGCGGGACATACTG
NDC1-R	CAACTATCCTCCAGCCCAAA
NUP210-F	ACGTGGTCGAACCTGGATAC
NUP210-R	TCACCAGGGTGAACAGTGAA
NUP153-F	TCAGGCCAAAAGAGAAAAGG
NUP153-R	TGGCTTTGGGGTCATAAGTC
TPR-F	GCGGCTTGAGCTAGAGAAAC
TPR-R	CAATATTGCGATCCTGAGCA
NUP50-F	CAAGTTGGGCAGGACTCATC
NUP50-R	GCGCTTTGCTTTCTTTATGG
GAPDH-F	GTCAGTGGTGGACCTGACCT
GAPDH-R	AGGGGTCTACATGGCAACTG
