# Supplementary figures and images for: Discovery of a Novel Aminocyclopropenone Compound That Inhibits BRD4-Driven Nucleoporin NUP210 Expression and Attenuates Colorectal Cancer Growth
Source: Cells. 2022 Jan 18;11(3):317. doi: 10.3390/cells11030317 (PMC8833887; doi:10.3390/cells11030317)

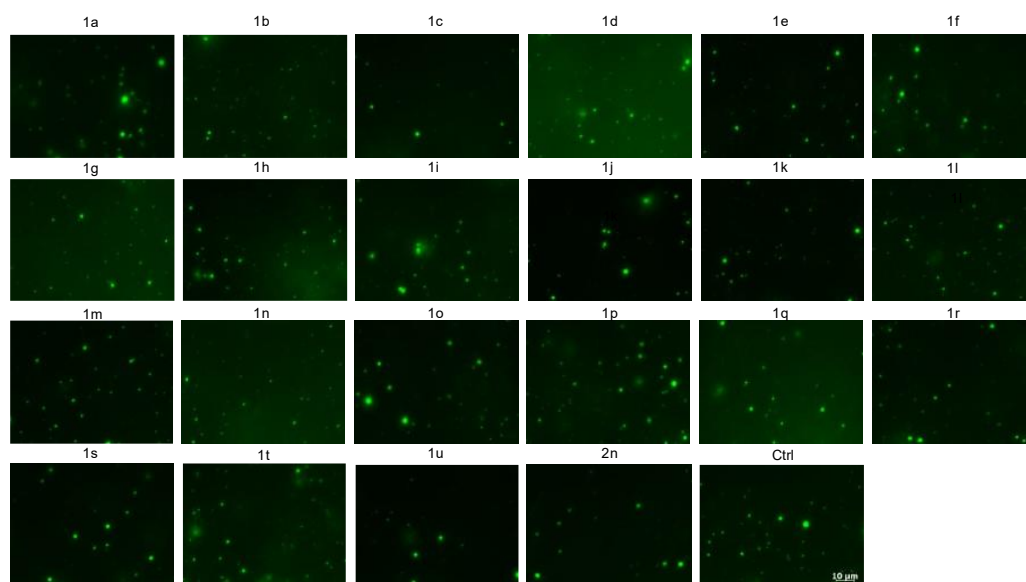

**Figure S2.** Representative BRD4 droplets pictures after each aminocyclopropanone treatment.

Supplement: Supplementary file 1 [file cells-11-00317-s001.zip › Supplementary Figure S2.pdf]
